# To adapt or go extinct? The fate of megafaunal palm fruits under past global change

**DOI:** 10.1098/rspb.2018.0882

**Published:** 2018-06-13

**Authors:** Renske E. Onstein, William J. Baker, Thomas L. P. Couvreur, Søren Faurby, Leonel Herrera-Alsina, Jens-Christian Svenning, W. Daniel Kissling

**Affiliations:** 1 Institute for Biodiversity and Ecosystem Dynamics (IBED), University of Amsterdam, PO Box 94248, 1090 GE Amsterdam, The Netherlands; 2 Royal Botanic Gardens, Kew, Richmond, Surrey, UK; 3 IRD, DIADE, Univ. Montpellier, Montpellier, France; 4 Department of Biological and Environmental Sciences, University of Gothenburg, Box 461, 405 30, Göteborg, Sweden; 5 Gothenburg Global Biodiversity Centre, Box 461, 405 30 Göteborg, Sweden; 6 Groningen Institute for Evolutionary Life Sciences, University of Groningen, Groningen, The Netherlands; 7 Center for Biodiversity Dynamics in a Changing World (BIOCHANGE), Department of Bioscience, Aarhus University, Aarhus, Denmark; 8 Section for Ecoinformatics and Biodiversity, Department of Bioscience, Aarhus University, Aarhus, Denmark

**Keywords:** biodiversity, extinction, frugivory, global change, megafauna

## Abstract

Past global change may have forced animal-dispersed plants with megafaunal fruits to adapt or go extinct, but these processes have remained unexplored at broad spatio-temporal scales. Here, we combine phylogenetic, distributional and fruit size data for more than 2500 palm (Arecaceae) species in a time-slice diversification analysis to quantify how extinction and adaptation have changed over deep time. Our results indicate that extinction rates of palms with megafaunal fruits have increased in the New World since the onset of the Quaternary (2.6 million years ago). In contrast, Old World palms show a Quaternary increase in transition rates towards evolving small fruits from megafaunal fruits. We suggest that Quaternary climate oscillations and concurrent habitat fragmentation and defaunation of megafaunal frugivores in the New World have reduced seed dispersal distances and geographical ranges of palms with megafaunal fruits, resulting in their extinction. The increasing adaptation to smaller fruits in the Old World could reflect selection for seed dispersal by ocean-crossing frugivores (e.g. medium-sized birds and bats) to colonize Indo-Pacific islands against a background of Quaternary sea-level fluctuations. Our macro-evolutionary results suggest that megafaunal fruits are increasingly being lost from tropical ecosystems, either due to extinctions or by adapting to smaller fruit sizes.

## 1. Introduction

**Figure 1.**
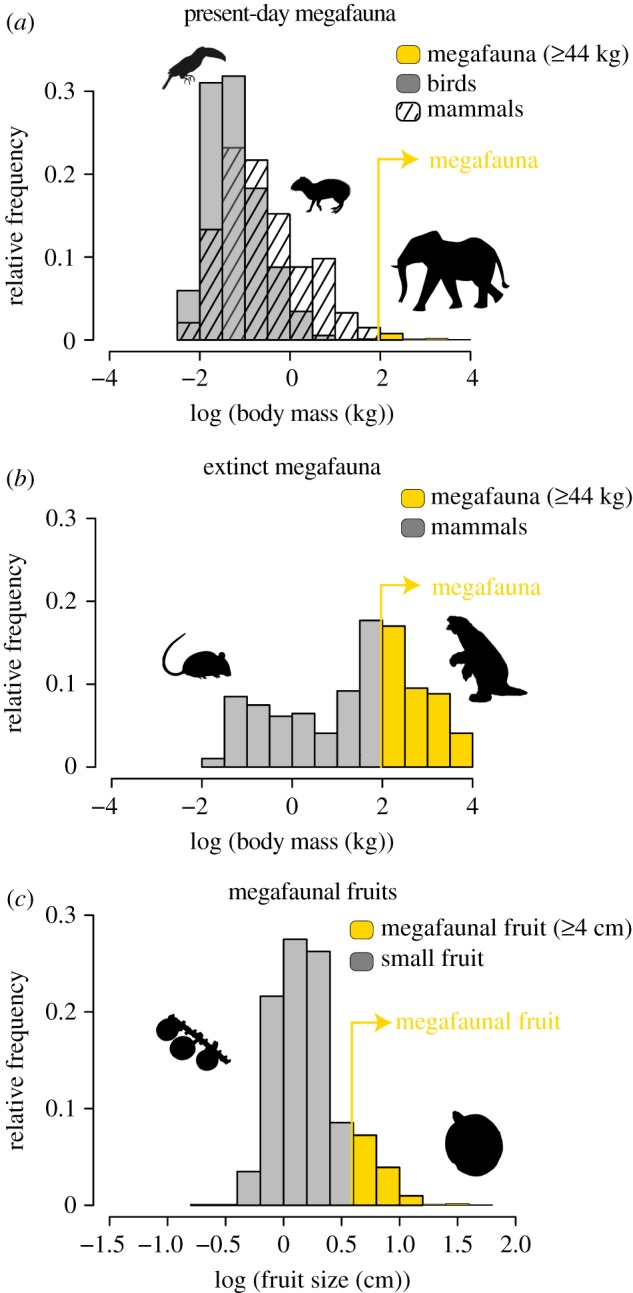
Global frequency distributions of megafaunal body sizes and megafaunal fruits. (*a*) Body size frequency distribution of present-day megafauna (*n* = 37 frugivorous mammals) compared with non-megafauna (*n* = 3726 frugivorous birds, *n* = 1645 frugivorous mammals). (*b*) Body size frequency distribution of extinct megafauna (*n* = 157 frugivorous mammals) compared with other extinct mammals (*n* = 137 frugivorous mammals). (*c*) Fruit size frequency distribution of palms (Arecaceae) with megafaunal fruits (*n* = 229 species) compared with palms with small fruits (*n* = 1607 species). (Online version in colour.)

Frugivory is a key plant–animal interaction. In tropical forests, more than 70% of woody plant species depend on frugivores for their seed dispersal [1]. In return, frugivores obtain essential nutrients from the fruits they consume [2]. The relationship between fruit size and animal body size (e.g. gape width and body mass) is a crucial factor for this mutualism. Large-bodied frugivores generally have large gape widths that enable them to ingest large fruits [1,3,4]. In particular, plants with megafaunal fruits—specifically, fruits ≥4 cm with a single (or few) large seed(s) [5,6]—depend on large-bodied mammals for their seed dispersal. These large-bodied mammals (e.g. tapirs, elephants and extinct proboscideans), often referred to as ‘megafauna’, are important for long-distance seed dispersal [7] and include mammals with body weights ≥44 kg [8]. This megafauna is nowadays rare (figure 1*a*), but has been common in the past (figure 1*b*). However, to what extent extinctions of megafauna have affected the present-day frequency distributions of fruit sizes in tropical plants, such as palms (figure 1*c*), remains unclear.

Extinctions of frugivorous megafauna may have severe consequences for the persistence of plant populations with megafaunal fruits [9–11]. Loss of megafauna leads to reduced dispersal and seedling recruitment, small geographical range sizes and loss of genetic variation [9,12–14]. This may ultimately lead to the extinction of plants with megafaunal fruits [14,15]. However, co-extinctions are not easily observed, especially in long-lived taxa, and the specific consequences of megafauna extinctions remain debated [5,6,16]. Furthermore, there may be a time lag between extinction cause, local extirpations and global extinction [17]. Specifically, it may take more than 25 000 years for plants with relatively large seeds to go extinct, following disperser extinctions [10]. Apart from local studies investigating population-level extinctions, information on deep-time extinctions of plant taxa with megafaunal fruits remains scarce.

Plants with megafaunal fruits may not only go extinct, but could also adapt to dispersal by small-bodied frugivores (e.g. by evolving smaller fruits and smaller seeds [18]). For example, the extinction of large-bodied frugivorous birds such as toucans, toucanets and large cotingas in some fragments of the Atlantic forest of Brazil have caused a consistent trend towards small seed sizes in a species of palm (*Euterpe edulis*) [18]. Although this adaptive change happened on short evolutionary time scales (i.e. decades), such micro-evolutionary processes may also leave signatures on macro-evolutionary, multimillion year time scales [19]. For instance, trait transition rates—the evolutionary change from one state to another within a trait—may reflect such adaptive changes on macro-evolutionary time scales. Increased transition rates have been observed in various plant lineages (e.g. to succulent and C_4_ plant syndromes in cacti, ice plants, agaves and grasses [20]). Such transition rate changes suggest that changes in environmental conditions such as the Late Miocene global expansion of arid environments and lowered atmospheric CO_2_ may have caused selective pressure for the evolution and adaptation of these trait syndromes [20]. However, deep time selection pressures for the evolution of megafaunal fruits remain controversial because several factors, such as climate, growth form and frugivory, may be associated with it [21].

Past (e.g. Cenozoic) global change has led to extinction, speciation and turnover of both taxonomic and functional diversity [22], causing major changes in biodiversity at local, regional and global scales [23–25]. This may have had severe consequences for biotic interactions such as animal-mediated seed dispersal and frugivory [26]. The Quaternary epoch, from 2.6 million years ago (Ma) to the present, has been characterized by episodes of rapid environmental changes, including periodical changes in temperatures and CO_2_ concentrations [27] and oscillating sea levels associated with glacial cycling [28]. This may have led to repeated fragmentation of vegetation and habitats, such as the expansion of dry-adapted vegetation at the cost of tropical rainforests, or the formation, connection and disconnection of islands worldwide [29,30]. These changes undoubtedly caused genetic divergences and extinctions via splitting and merging of gene pools [31], thereby impacting plant and animal distributions and biodiversity [32–34]. Examples are severe extinctions of frugivorous megafauna, such as gomphotheres, ground sloths and glyptodonts in the Late Quaternary [6,8]. The Quaternary epoch could therefore be a crucial time period to evaluate the consequences of historical global change for the relative importance of extinction versus adaptation of vertebrate-dispersed tropical plant lineages. However, selective extinctions of forest-adapted megafauna (notably browsers and frugivores) in various New World and Old World regions in response to late Cenozoic cooling and drying may also have happened much earlier (i.e. from the Miocene onwards [35]).

Here, we investigate the macro-evolutionary dynamics of extinction and adaptation of megafaunal fruits at large spatial (biogeographic) and temporal (late Cenozoic) scales. We test two hypotheses. First, we hypothesize that the recurrent and rapid climatic shifts during the Quaternary have led to an increase of extinction rates of plants with megafaunal fruits (H1). This is based on the expectation that megafaunal fruits will be exposed to increasing dispersal limitation due to repeated fragmentation of vegetation and habitats and megafauna extinctions [13–15,27]. Second, we hypothesize an increase in transition rates from megafaunal to small fruits, indicative of repeated, parallel evolution of small fruits (H2). This might be driven by the need to disperse with volant frugivores, especially in insular environments where birds and bats are more successful dispersers than mammalian megafauna in tracking rapid climatic and sea-level changes [28,36]. We test these two hypotheses in palms (Arecaceae), a keystone plant family for vertebrate frugivores in the tropics [2]. The palm family is globally distributed and comprises around 2600 species, of which the majority is restricted to rainforest habitats [37,38]. Their seeds are dispersed by a wide range of frugivores, primarily birds and mammals (but also reptiles and fish) [37,39]. Using fruit sizes for 70% of the palm species combined with global species distribution data [40] and a comprehensive species-level phylogeny for all palms [41], we fit diversification rate models in a time-window analysis over the late Cenozoic (i.e. last 25 million years). This allows us to investigate the evolutionary fate of palm lineages with megafaunal fruits (≥4 cm in length) [5,6], compared with those with small fruits (less than 4 cm length).

## 2. Results

### (a) Evolution of megafaunal palm fruits

**Figure 2.**
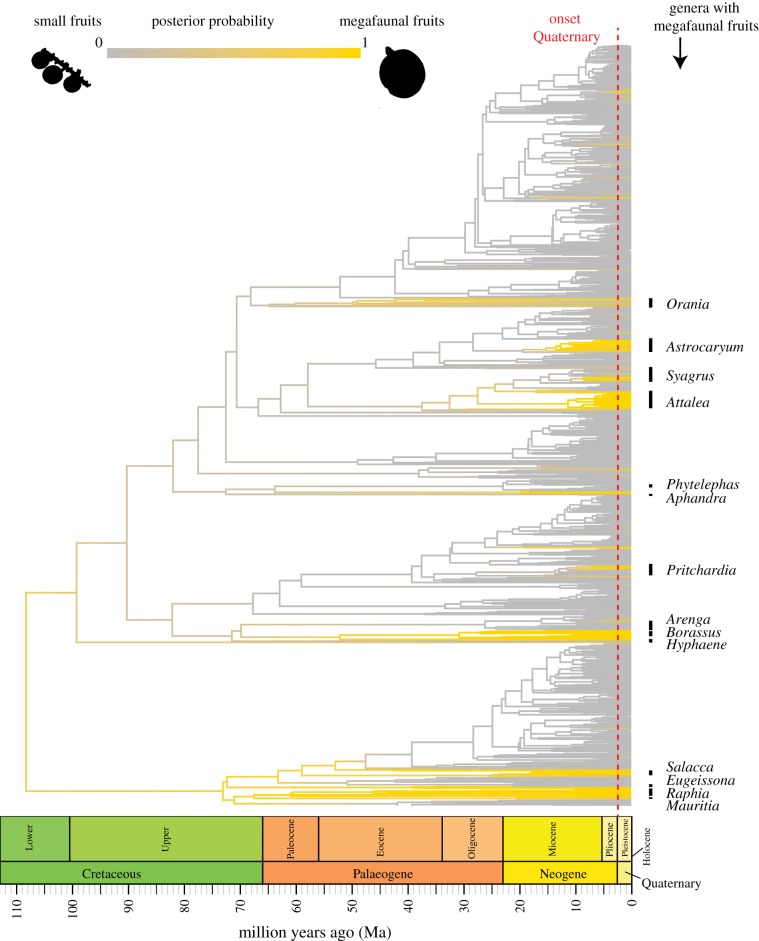
Macroevolution of megafaunal palm fruits (Arecaceae). The palm phylogeny shows the posterior probability of megafaunal fruits (yellow) at the internal branches and nodes on the maximum clade credibility (MCC) tree. Note that the MCC tree is just for illustration purposes (all analyses were performed on 100 randomly selected palm phylogenies from the posterior distribution). The probabilities were derived from ancestral state reconstructions under 500 stochastic character maps. The reconstruction suggests that all palm fruits have evolved from the ancestral state of a megafaunal palm fruit (*ca* 110 Ma). Examples of palm genera that comprise at least one species with megafaunal fruits are indicated at the tips (for the full list see electronic supplementary material, figure S4). Megafaunal fruits ≥4 cm length. All other palms with small fruits less than 4 cm in length. (Online version in colour.)

By compiling a comprehensive fruit trait dataset, we found that 12% (*n* = 227) of all measured palms (*n* = 1834) produce megafaunal fruits of ≥4 cm length (figure 1*c*). Megafaunal fruits are, relative to small fruits (less than 4 cm length), more common in the New World (*n* = 110 species, 16%) than in the Old World (*n* = 117 species, 10%). Examples of typical megafaunal fruits are found in the New World genera *Mauritia*, *Aphandra* and *Phytelephas*, and in the Old World genera *Raphia*, *Eugeissona*, *Hyphaene* and *Arenga* (figure 2; see also electronic supplementary material, table S1 for an overview of the biogeographic distribution of palm genera and their fruit sizes). Our reconstruction of ancestral trait states (figure 2) suggests that all palm fruits originated from a palm ancestor with megafaunal fruits (*ca* 110 Ma). Several lineages with megafaunal fruits, such as *Raphia*, *Salacca* and *Borassus*, probably retained the ancestral megafaunal fruit state without any transitions to smaller fruits. Other lineages, such as *Pritchardia*, *Attalea*, *Syagrus*, *Astrocaryum* and *Orania*, seem to have regained megafaunal fruits after a previous transition to small fruits (figure 2).

### (b) Late Cenozoic extinction rates

To test the hypothesis (H1) that extinction rates of palm lineages with megafaunal fruits have shifted in response to Quaternary climate change, we used time-dependent diversification models in a Bayesian time-slice analysis (see Methods). These models were fitted to the phylogenetic dataset with temporal cut-offs at 25, 20, 15, 10, 5, 2.6, 1 and 0.5 Ma, covering the Neogene and Quaternary.

**Figure 3.**
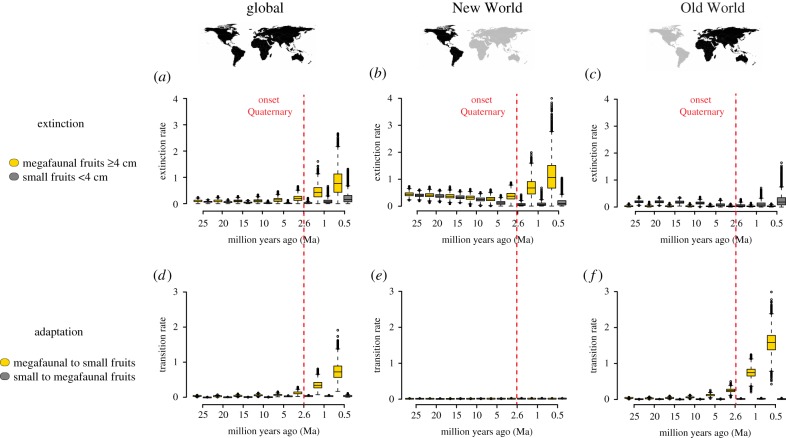
Late Cenozoic extinction and adaptation dynamics of palm lineages with megafaunal fruits. Shown are extinction rates (*a–c*) and transition rates (*d–f*) for global, New World and Old World palms. Both extinction and transition rates are estimated through Markov chain Monte Carlo on 100 randomly selected palm phylogenetic trees from the posterior distribution for 10 000 generations, given the selected diversification models (see electronic supplementary material, tables S6–S8). Box-and-whiskers indicate the median, quartiles (25% and 75%), minimum (5%), maximum (95%) and outliers of these rates for each time slice over the last 25 Myr. The onset of the Quaternary epoch (2.6 Ma) is indicated in red. Extinction rates of palms with megafaunal fruits increase globally and in the New World whereas transition rates (evolving smaller fruits from megafaunal fruits) increase globally and in the Old World. (Online version in colour.)

The geographical regions (global, New World and Old World) showed different results with respect to extinction rates (figure 3*a–c*). Globally, we detected an exponential increase in extinction rates of palm lineages with megafaunal fruits from the onset of the Quaternary (figure 3*a*). In contrast, palms with small fruits did not show such an increase (figure 3*a*). This global result was also reflected in the New World. The extinction rate of palm lineages with megafaunal fruits in the New World (figure 3*b*) was estimated to be 10-fold the extinction rate of New World palm lineages with small fruits during the last 0.5 Myr. In contrast, extinction rates of New World palm lineages with small fruits showed a moderate decrease during the late Cenozoic (figure 3*b*). In the Old World, we did not detect any temporal shift in extinction rates for palm lineages with either small or megafaunal fruits (figure 3*c*).

### (c) Late Cenozoic transition rates

The geographical regions (global, New World and Old World) also showed differences in transition rates (H2, figure 3*d–f*). Globally, an exponential increase in transition rates of megafaunal fruits (i.e. from megafaunal to small fruits) was detected from the onset of the Quaternary onward (figure 3*d*). In contrast, transition rates from small to megafaunal fruits remained relatively low and constant throughout the late Cenozoic (figure 3*d*). In the New World, no such transition rate increase for palms with megafaunal fruits was detected: both palm lineages (with small and megafaunal fruits, respectively) showed constant rates of transition to megafaunal and small fruits during the late Cenozoic (figure 3*e*). However, patterns in the Old World were consistent with the global result. Here, we detected an exponential increase in transition rates from megafaunal to small fruits from the onset of the Quaternary onward (figure 3*f*). These Old World rates currently exceed 100-fold the transition rates from small to megafaunal fruits. In contrast, the Old World transition rates from small to megafaunal fruits remained more or less constant during the late Cenozoic (figure 3*f*), similar to the global analysis. As 95% of the Old World palm species occur in the Indo-Pacific region (1083 out of 1139 sampled species), this signal is driven by diversification in these island-dominated environments, rather than by diversification on the relatively species-poor African continent. This was confirmed when removing the Afrotropical species (*n* = 56) from the analysis, providing qualitatively similar results.

## 3. Discussion

### (a) Evolution of megafaunal palm fruits

We investigated the fate of palms with megafaunal fruits during the late Cenozoic. Using a comparative phylogenetic approach and a time-window analysis, we show that from the onset of the Quaternary (2.6 Ma)—a period characterized by global cooling, drying and recurrent climatic oscillations [27]—New World palms with megafaunal fruits (≥4 cm in length) have experienced increasing extinction rates (figure 3*b*), whereas Old World palms with megafaunal fruits show increasing transition rates, evolving small fruits (less than 4 cm in length) from megafaunal fruits (figure 3*f*). In other words, Old World megafaunal palms appear to be adapting to global change, while those in the New World are dying out.

### (b) Late Cenozoic extinction rates

We detected increasing extinction rates of New World palms with megafaunal fruits from the onset of the Quaternary onward. The on-going late Cenozoic cooling and drying, and the recurrent climate oscillations, may have contributed to habitat fragmentation of rainforests (e.g. by wetlands [24,32]). Megafaunal fruits may be a handicap in such fragmented landscapes because they cannot be dispersed by volant frugivores which successfully move among forest fragments and can track climate changes rapidly [36]. Furthermore, rainforest fragmentation may have led to defaunation of megafauna, such as the progressive selective extinctions of forest-adapted browsers since the Miocene [35]. Both the fragmentation of rainforest habitat and the defaunation of megafauna may have led to disrupted dispersal of palms with megafaunal fruits, ultimately triggering their extinction.

Our detected extinction rate increase in the Quaternary was preceded by the invasion of North American placental carnivores into South America during the Great American Biotic Interchange (*ca* 3 Ma). These carnivores may have caused extinctions among medium-sized South American frugivores, such as ‘small’, deer-sized megafauna and ground-living birds [42]. Therefore, they may have caused a temporary decrease in megafauna diversity in the initial stages of this event [25]. These carnivore invasions and the subsequent Late Quaternary extinctions (14 000–7000 years ago) of frugivorous megafauna [6,8] (figure 1*b*) may have exacerbated extinctions of palms with megafaunal fruits through dispersal limitation and the reduction of range sizes [10]. Palynological data confirm local Quaternary extinctions of Neotropical palms with megafaunal fruits (e.g. in the genus *Mauritia* [43]). The Andean uplift, Quaternary climate change and the progressive fragmentation of palm populations could be the cause of these extinctions [43].

Inferring extinction rates from molecular phylogenies remains challenging and controversial [44–46]. This is also true for our estimates of extinction rates, which show some uncertainty for New World palms with megafaunal fruits (electronic supplementary material, figures S1 and S2). Unfortunately, reliable information on palm extinctions from the fossil record remains scarce [37]. Nevertheless, future work that combines the available fossil record with phylogenies may improve our understanding of past extinction events.

### (c) Late Cenozoic transition rates

Old World palms did not show an accelerated extinction rate as detected in the New World, but instead an increase in transition rates from megafaunal to small fruits from the onset of the Quaternary onward. Our results are congruent with previous findings showing a macroevolutionary trend towards smaller fruit sizes in animal-dispersed Sapindales lineages in the Indo-Malay Archipelago [47]. Although changes in fruit and seed sizes have been common across angiosperms and geological times [21,48], the selection pressures for such evolutionary changes remain controversial. For instance, adaptive changes of fruit and seed sizes have been associated with climate change, recruitment chances and growth forms rather than with frugivory [21]. Nevertheless, selection pressures of frugivores over macroevolutionary time scales have yet been rarely studied and could provide an important explanation, especially because megafauna may exert a limit to maximum fruit size [21,47,49].

We suggest that an increase in transition rates to small fruits over millions of years may reflect adaptive changes to an increase in seed dispersal by medium-sized, strong-flying volant frugivores such as birds and bats. This may have happened especially in the archipelagic setting of the Indo-Pacific region, with its complex tectonic history [30] and Quaternary sea-level changes [34]. This dynamic island-dominated environment may have selected for smaller fruits because these provide an advantage for the successful colonization of new areas [2,50]. Furthermore, small fruits may provide an advantage over megafaunal fruits to track climate and sea-level changes [36]. The Indo-Pacific harbours a high diversity of strong-flying, ocean-crossing frugivores such as fruit pigeons (Columbiformes), hornbills (Bucerotiformes) and fruit bats (Pteropodidae) [51]. These animals may have facilitated palm dispersals across islands, thereby reinforcing the selection for small (less than 4 cm) bird- and bat-dispersed palm fruits, and also favouring their increased diversification [52].

## 4. Conclusion

The inferred macro-evolutionary rates of palms suggest that global changes in the Quaternary—including temperature oscillations, sea-level fluctuations and habitat fragmentation—have likely altered the coevolutionary dynamics between fruits and frugivores [25,53]. Specifically, the progressive loss of frugivores in response to Late Cenozoic cooling and drying together with climatically driven habitat fragmentation of rainforests and the subsequent defaunation of megafauna may have strongly distorted plant–frugivore interactions in the New World, resulting in increased extinction rates of palms with megafaunal fruits. Moreover, an increased selection for seed dispersal by medium-sized, strong-flying, ocean-crossing frugivores may explain the increase in transition rates from megafaunal to small palm fruits, especially in the island-dominated environments of the Indo-Pacific against a background of Quaternary climate and sea-level changes. Our results suggest that plants with megafaunal fruits may be increasingly lost from ecosystems. This may have far-reaching consequences for ecosystem processes, including carbon storage in tropical forests [54].

## 5. Methods

### (a) Palm data

We used previously published phylogenetic data [41], which includes all 2539 palm species. All analyses were performed on 100 phylogenetic trees from the posterior distribution. For details on the phylogenetic inference, see electronic supplementary material.

Fruit lengths for 1834 vertebrate-dispersed palm species were collected from published literature and were updated to the latest palm taxonomy. Species were classified into two main groups: small-fruited (less than 4 cm in length) and large, megafaunal-fruited (≥4 cm in length) palms [5,6]. This classification was based on the seed dispersal ecology of the species and directly follows the classification of megafaunal fruits by Guimarães *et al.* [5]. We note that we used fruit length rather than fruit diameter because data on fruit diameter were unavailable for 405 (out of 1834) palm species, and because fruit length strongly correlates with fruit diameter (see electronic supplementary material, figure S3). Palm species with megafaunal fruits rely on large animals (megafauna ≥44 kg) such as tapirs, elephants and extinct gomphotheres, ground sloths and glyptodonts for their seed dispersal, whereas palm species with small fruits are predominantly dispersed by birds, bats and non-volant, smaller-bodied mammals (figure 1). Dispersal by these different frugivore ‘guilds’ is expected to have contrasting effects on past extinction and transition rates of palms, thereby providing a solid comparative framework. Nevertheless, several palms (particularly in subtribe Attaleinae) have very large, nut-like fruits without fleshy pulp [37]. Nowadays, these species rely on dispersal by rodents rather than megafauna and may therefore not bear truly ‘megafaunal’ fruits [5]. Although this fruit type could not be distinguished in our database, we evaluated the impact of this trait on the results by repeating the analyses excluding the Attaleinae (see sensitivity analyses below for details).

We used a world checklist of palms [40] to assign species to the New World (the Americas [predominantly Neotropics] and Caribbean islands) and Old World (Africa, tropical Asia, Australasia, and the Pacific). This classification reflects the strong dispersal limitation of palms that has led to a high degree of palm endemism in these regions [23,55] (electronic supplementary material, table S1), suggesting largely independent evolutionary histories of New World and Old World palms. For more details on fruit trait and distribution sampling, see electronic supplementary material.

### (b) Frugivore data

To generate relative frequency plots of frugivore guilds and their body sizes (figure 1), we assembled body size and diet data on extant and extinct mammalian and avian frugivores. For more details see electronic supplementary material.

### (c) Ancestral state reconstructions

We sampled 500 stochastic character maps of ancestral fruit sizes to evaluate the posterior probability of ancestral megafaunal fruits at the internal branches and nodes of the palm phylogenetic tree (figure 2). These ancestral state reconstructions gave qualitatively similar results to those when we used the parameters (electronic supplementary material, table S3) from the global Binary State Speciation and Extinction (BiSSE) model (for details see below) to reconstruct marginal ancestral states for megafaunal fruits at the internal nodes (for more details see electronic supplementary material, figure S4).

### (d) Simulations on trait-dependent diversification

The BiSSE model [56,57] implemented in the ‘diversitree’ R package [58] was used to model speciation, extinction and transition rates of palm lineages with small versus megafaunal fruits. Recent criticism on trait-dependent diversification models [59] has encouraged researchers to perform simulations to test for type I and type II error rates in the data. We therefore performed three simulation studies.

First, we randomly evolved a neutral binary trait on 10 empirical palm phylogenies under four transition rate (‘q’) scenarios (*q* = 0.01, *q* = 0.1, *q* = 1 and *q* = 10) (following suggestions by ref. [59]), and also used our observed transition rates from the global dataset (*q*_megafaunal to small_ = 0.017; *q*_small to megafaunal_ = 0.006) on the simulation of a neutral trait on 100 empirical palm phylogenetic trees. Second, we simulated 10 birth–death trees with 1774 tips (the sample size in the empirical trees) of age 105 Ma (the age of palms) under relatively high extinction rates (speciation = 0.2, extinction = 0.19), creating trees with similar tree shapes to the empirical phylogenetic trees (see lineage through time plots in electronic supplementary material, figure S5) and randomly evolved neutral traits with equal transition rates on these trees (*q* = 0.02). Third, we used the trait-dependent diversification process to simultaneously evolve 10 phylogenetic trees and a binary trait. First, we simulated an extinction rate shift at 2.6 Ma for one of the trait states (extinction rate from 0.02 to 0.3), whereas the other trait state kept the same extinction rate. Similarly, we simulated a transition rate shift at 2.6 Ma for one of the trait states (transition rate from 0.005 to 0.34), whereas the other trait state kept the same transition rate. These simulations were done to test whether our data have the power to correctly infer an increase in extinction or transition rates for one of the trait states in the Quaternary when it is truly there (for more details on these methods and simulations see electronic supplementary material).

For the first simulation study, and concerning the extinction rates when simulating neutral traits under low transition rate scenarios (*q* = 0.01 or *q* = 0.1) as well as under the observed, empirical transition rate parameters, we detected increasing extinction rates in the late Quaternary similar to the empirical results (electronic supplementary material, figures S1*b*,*f* and S2*b*). These results suggest that the increasing extinction rate of global and New World palms with megafaunal fruits from the onset of the Quaternary onwards (figures 3*a*,*b*) may be partly explained by the shape of the phylogenetic tree (i.e. the distribution of branch-lengths), rather than fruit sizes [59]. The results on extinction rates should therefore be taken with caution. However, under high transition rate scenarios (*q* = 1 or *q* = 10) neutral traits did not show this increasing extinction rate (electronic supplementary material, figure S1*j*,*n*), potentially confirming the reliability of the empirical results. Concerning the transition rates, neutral trait simulations under all transition rate scenarios confirmed that there was a lack of increase in transition rates from the onset of the Quaternary (electronic supplementary material, figures S1*d*,*h*,*l*,*p* and S2*d*), as would be expected under neutral trait evolution. This suggests that the empirical transition rate estimates that we detected for global and Old World palms with megafaunal fruits (figures 3*d*,*f*) are robust with respect to the palm phylogenetic tree shape.

For the second simulation study, we did not detect any Quaternary increases in extinction or transition rates of neutral traits on our simulated birth-death trees (electronic supplementary material, figure S6). This suggests that the imbalance in tree shape, number of lineages and splitting events between the time slices (see electronic supplementary material, table S2) does not influence the inference of extinction or transition rates.

For the third simulation study, we were able to correctly infer an increase in extinction and transition rates for one of the trait states from 2.6 Ma onward, suggesting that the time-dependent BiSSE model is able to infer these rates correctly when they are modelled to be there (electronic supplementary material, figure S7). However, when modelling a transition rate shift, the extinction rate may also erroneously increase, although this erroneous correlation was not observed in the empirical data: New World palms with megafaunal fruits showed increased extinction rates without also increasing their transition rates (figure 3*b*,*e*). In other words, the increased extinction in New World palms may be a true phenomenon, as it does not occur as an effect of increased transition rates.

By including all extant palm species and their traits in a phylogenetic framework we were able to detect general, historical patterns. This dataset is therefore robust to issues related to biased sampling of taxa or traits (i.e. 12% of palm species have megafaunal fruits) [60]. Furthermore, by including all palms we maximized the sample size that is required to estimate the parameters in diversification rate models [56]. This also maximizes the number of independent, evolutionary events and thereby avoids pseudo-replication [61]. This approach may thus be applicable to other well-sampled clades for which (near-)complete phylogenetic and functional trait data are available.

### (e) Trait-dependent diversification rates

To test the hypotheses that extinction and transition rates of palm lineages with small versus megafaunal fruits have shifted in response to global environmental change, we used a time-dependent BiSSE model. This was done at the global level and within the New World and Old World separately. We performed our initial model selection by using a 2.6 Ma cut-off value (*t* = 2.6), but repeated the Bayesian analyses (see below) with temporal cut-offs at 25, 20, 15, 10, 5, 1 and 0.5 Ma, covering the Neogene and Quaternary. For all three geographical regions (global, New World and Old World), a time-dependent model in which speciation, extinction and/or transition rates of palm lineages showed a significant shift during the Quaternary fitted better than a constant rate (i.e. time independent) model (see electronic supplementary material, tables S3–S5 for model selection).

We then used a step-wise model selection approach to fit up to 43 diversification models to the global (electronic supplementary material, table S6), New World (electronic supplementary material, table S7) and Old World (electronic supplementary material, table S8) datasets (see electronic supplementary material, figure S8 for an overview of the parameters in these models). These models contained different combinations of constrained and free parameters (i.e. speciation, extinction and/or transition rates were constrained to be equal for lineages with small versus megafaunal fruits, and/or for lineages evolving pre-Quaternary and Quaternary, or they were allowed to differ freely; electronic supplementary material, figure S8). We compared these models using likelihood-ratio tests (nested-models) and the Akaike information criterion (AIC) (non-nested models), and selected the best-fitting models given the fewest number of parameters without significantly affecting model-fit (ΔAIC < 2). Sampling fractions reflecting species and their traits (small or megafaunal fruits) sampled from the total were used (32% of species with small fruits and 18% of species with megafaunal fruits were not sampled in the global dataset). Sampling fractions were corrected for the New World and Old World datasets. A Bayesian Markov chain Monte Carlo (MCMC) was run for the best-fitting model for 10 000 generations on 100 randomly sampled palm phylogenies. We plotted the posterior distributions (95% Bayesian credibility intervals) of the parameter estimates for the extinction and transition rates (figure 3), and the speciation and net diversification rates (electronic supplementary material, figure S9) through time.

### (f) Sensitivity analyses

We performed two sensitivity analyses to assess the robustness of our results with respect to the classification of palms with megafaunal fruits. First, we repeated the time-dependent diversification analyses using cut-off values of ≥3.5 cm and ≥4.5 cm to classify palm species with megafaunal fruits (*n* = 260 for 3.5 cm analysis; *n* = 186 for 4.5 cm analysis) versus small fruits (*n* = 1514 for 3.5 cm analysis; *n* = 1588 for 4.5 cm analysis). Second, we repeated the time-dependent diversification analyses excluding species in the subtribe Attaleinae (*n* = 98), which have predominantly nut-like fruits (for more details on these methods see electronic supplementary material).

Both sensitivity analyses show qualitatively similar results to our original analysis (i.e. increasing extinction rates of palm lineages with megafaunal fruits and increasing transition rates from megafaunal to small fruits during the Quaternary; compare electronic supplementary material, figures S10 and S11 with figure 3). These results suggest that our classification of palms with megafaunal fruits ≥4 cm, and the inclusion of palms with large, nut-like fruits, are not driving the overall diversification patterns obtained in this study.

## Supplementary Material

Onstein_et_al_methods_tables_figures_ESM_R2

## Supplementary Material

ReadMe

## Supplementary Material

functions_for_simulation.R

## Supplementary Material

main_runSimulation.R

## Data accessibility

The phylogenetic data and the fruit size data that support the findings of this study are available from the Dryad Digital Repository: http://dx.doi.org/10.5061/dryad.cm4nm. The palm species distribution data are available from the World Checklist of Selected Plant Families (http://apps.kew.org/wcsp). The R script and program to perform the simulations are available from the electronic supplementary material. Additional data files are available from the authors on request.
