# Supplementary material for: To adapt or go extinct? The fate of megafaunal palm fruits under past global change
Source: Proc Biol Sci. 2018 Jun 13;285(1880):20180882. doi: 10.1098/rspb.2018.0882 (PMC6015859; doi:10.1098/rspb.2018.0882)

Supplementary Information

To adapt or go extinct? The fate of megafaunal palm fruits under past global change

Renske E. Onstein[1,*], William J. Baker[2], Thomas L. P. Couvreur[3], Søren Faurby[4,5], Leonel Herrera-Alsina[6], Jens-Christian Svenning[7,8] & W. Daniel Kissling[1]

Published in Proceedings of the Royal Society B

[1]Institute for Biodiversity and Ecosystem Dynamics (IBED), University of Amsterdam, P.O. Box 94248, 1090 GE Amsterdam, The Netherlands.
[4]Department of Biological and Environmental Sciences, University of Gothenburg, Box 461, SE 405 30, Göteborg, Sweden.
[5]Gothenburg Global Biodiversity Centre, Box 461, SE 405 30 Göteborg, Sweden.
[2]Royal Botanic Gardens, Kew, Richmond, Surrey, UK.
[3]IRD, DIADE, Univ. Montpellier, Montpellier, France.
[6]Groningen Institute for Evolutionary Life Sciences, University of Groningen, Groningen, The Netherlands.
[7]Center for Biodiversity Dynamics in a Changing World (BIOCHANGE), Aarhus University, Aarhus, Denmark.
[8]Section for Ecoinformatics and Biodiversity, Department of Bioscience, Aarhus University, Aarhus, Denmark.

*Corresponding author: Renske E. Onstein, Institute for Biodiversity and Ecosystem Dynamics (IBED), University of Amsterdam, Amsterdam, The Netherlands. Tel: +31(0)646583916, e-mail: onsteinre@gmail.com.

Content

Supplementary methods 3
Supplementary tables 9
Supplementary figures 23

Details:

Supplementary tables
Table S1: Biogeography of palm fruit sizes.
Tables S2: Summary of number of speciation events, number of lineages, transition rates and number of transitions for each time slice in this study.
Tables S3–S5: Trait-dependent vs. time-dependent diversification model selection for global, New World and Old World palms.
Tables S6–S8: Trait- and time-dependent diversification model selection for global, New World and Old World palms.

Supplementary figures
Figure S1: Posterior densities (box-and-whiskers) of time-dependent speciation, extinction, transition and net diversification rates for global palms when binary traits are simulated under several transition rate scenarios (simulation).
Figure S2: Posterior densities (box-and-whiskers) of time-dependent speciation, extinction, transition and net diversification rates for global palms when binary traits are simulated under the observed, empirical transition rate scenarios (simulation).
Figure S3: The relationship between fruit length and fruit diameter in palms.
Figure S4: Ancestral megafaunal palm fruit reconstructions under the BiSSE parameters.
Figure S5: Lineage through time plots of 100 empirical palm and simulated birth-death phylogenies.
Figure S6: Posterior densities (box-and-whiskers) of time-dependent speciation, extinction and transition rates for simulated binary traits on simulated birth-death phylogenetic trees (simulation).
Figure S7: Posterior densities (box-and-whiskers) of time-dependent speciation, extinction and transition rates for the simulated trait-dependent diversification process in which extinction and transition rates for one of the trait states shift at 2.6 Ma (simulation).
Figure S8: Parameters in the time-dependent diversification models.
Figure S9: Posterior densities (box-and-whiskers) of time-dependent speciation and net diversification rates for global, New World and Old World palms (empirical).
Figure S10: Posterior densities (box-and-whiskers) of time-dependent extinction and transition rates for global palms when species with megafaunal fruits are classified as having fruits ≥ 3.5 cm or ≥ 4.5 cm (empirical).
Figure S11: Posterior densities (box-and-whiskers) of time-dependent extinction and transition rates for global palms, excluding subtribe Attaleinae (empirical).

Supplementary methods

Phylogenetic data

The phylogenetic data includes 2539 palm species (which were all accepted species at the time of publication) and is based on a backbone generated from nine plastid and four nuclear markers, morphological data from ref. [1], supplemented with additional sequences for several genera [2], and dated following the five calibrations described by ref. [3]. A Bayesian modeling approach was used to place species without genetic or morphological data in the phylogeny, based on an estimation of validity of intra-generic taxonomic groupings as topological constraints [for details see ref. 2]. As this leads to uncertainty in the exact placement of a species within the tree, all analyses were performed on a set of 100 randomly sampled palm phylogenetic trees. For visualization purposes and model testing, a maximum clade credibility (MCC) tree was generated in TreeAnnotator v1.8.2 [4]. Although crown-group palms radiated initially in the Cretaceous ca. 110 Ma, most diversity emerged during the Cenozoic (65 Ma until present) (figure 2).

Fruit size data

Fruit lengths for 1834 vertebrate-dispersed palm species were collected from published literature and were updated to the latest palm taxonomy. In case multiple records per species were available, the species average was calculated. Species were classified into two main groups: small-fruited (< 4 cm in length) and large, megafaunal-fruited (≥ 4 cm in length) palms [5, 6] (also see summary statistics in table S1). This classification was based on the seed dispersal ecology of the species: large-fruited palm species rely on large animals (megafauna ≥ 44 kg) such as tapirs, elephants and extinct gomphotheres, ground sloths and glyptodonts for their seed dispersal, whereas small-fruited palm species are mainly dispersed by birds, bats and non-volant, smaller-bodied mammals (figure 1). Dispersal by these different frugivore 'guilds' is expected to have differently affected past extinction and transition rates of palms, thereby providing a valid comparative framework. We note that fruit length rather than fruit diameter was used in this study because data on fruit diameter were unavailable for 405 (our of 1834) palm species, and because fruit length strongly correlates with fruit diameter (see figure S3). Furthermore, several palms (particularly in subtribe Attaleinae) have very large, nutlike fruits without fleshy pulp and may rely on dispersal by rodents rather than megafauna. Although this fruit type was not distinguished in our database, we evaluated the impact of this trait on the results by repeating the analyses excluding the Attaleinae (see sensitivity analyses below for details).

For those species not sampled for fruit size in the phylogeny (n = 765) we imputed missing values based on the phylogenetic relationships between species and their trait values [using the phylopars command in the R 'phyloPars' package, ref. 7], and used these to test for biased sampling (see below for details; imputed trait values were not used directly in the analyses). These estimates suggested that we sampled 68% of palm species with small fruits and 82% of palms species with megafaunal fruits.

Distribution data

We used the world checklist of palms [8] (download from June 2015) to assign species to the New World (predominantly Neotropics) and Old World (Africa, tropical Asia, Australasia and Pacific). This classification reflects the strong dispersal limitation of palms that has led to

a high degree of palm endemism in these regions [9, 10] (table S1), suggesting largely independent evolutionary histories of New World and Old World palms. Moreover, these regions have responded differently to Quaternary global change. For example, South America experienced the Late Quaternary megafauna extinctions more severely [11], whereas the island-dominated environments of the Indo-Pacific region may have been particularly affected by Quaternary sea level oscillations [12].

Frugivore data

We used frugivore classifications of birds [13] and mammals [MammalDIET, 14] to identify extant species for which at least part of their diet consists of fruit (birds n = 3726; mammals n = 1682). We additionally extracted the body masses (kg) for these species from ref. [15] and ref. [16] and log-transformed them. For Quaternary extinct mammals, we extracted body mass [16] and diet data [17] resulting in data for n = 294 extinct mammals. Frugivores were classified as those including 'plants' in their diet. Frugivorous mammals ≥ 44 kg were classified as megafauna (extant mammals n = 37; extinct mammals n = 157). We excluded megafaunal birds (n = 2, Casuariidae). These data were used to evaluate the relative frequency of frugivores and their body sizes (figure 1).

Simulations on trait-dependent diversification

The Binary State Speciation and Extinction (BiSSE) model [18, 19] implemented in the 'diversitree' R package [20] was used to model speciation, extinction and transition rates of palm lineages with small vs. megafaunal fruits. The BiSSE model jointly estimates these rates by using dated phylogenetic trees in which branch-lengths reflect time, and binary trait states (i.e. small vs. megafaunal fruits) are assigned to the species at the tips of the phylogenetic tree. The joint-estimation of these rates is desirable as trait transitions may not be independent from speciation and extinction rates [19]. Maximum likelihood is used to optimize full and constrained models (see below) and Bayesian Markov chain Monte Carlo (MCMC) is subsequently used to evaluate uncertainty in the parameter estimates of the models used.

Recent criticism on trait-dependent diversification models [21] has encouraged researchers to perform simulations to test for type I (i.e. detecting a significant effect when it is not truly there) and type II (i.e. not detecting a significant effect when it is truly there) error rates. To assess the impact of these error rates using our data and methods, we performed three simulation studies.

First, we randomly evolved a neutral binary trait (with states A and B) on 10 empirical palm phylogenies under four transition rate ('q') scenarios (q = 0.01, q = 0.1, q = 1 and q = 10) [following suggestions by ref. 21], providing a gradient from rare to frequent character state changes. These simulated traits are expected to be 'neutral' with respect to speciation, extinction or transition rates, as they evolved under a simple 'Markov discrete' (Mk) [22] model of evolution. We then evaluated the changes in 95% Bayesian credible intervals in speciation, extinction and transition rates over time given the selected models from our initial analysis (see table S6), and after running the MCMC for 1000 generations (results in figure S1). We repeated this procedure using our observed transition rates from the global dataset ($q_{\text{megafaunal to small}}$ = 0.017; $q_{\text{small to megafaunal}}$ = 0.006) on the simulation of neutral traits on 100 empirical palm phylogenetic trees (results in figure S2).

Second, we simulated 10 birth-death phylogenetic trees of 1774 species (the sample size in the empirical trees) with an age of 105 Ma (the age of palms) with extinction rate = 0.19 and speciation rate = 0.2. This creates phylogenetic trees in which lineages accumulate through time similarly to our observed data (see figure S5). We then randomly evolved a

neutral binary trait (with states A and B) on these phylogenies under an equal transition rate scenario (q = 0.02). Again, we evaluated the changes in 95% Bayesian credible intervals in speciation, extinction and transition rates over time given the selected models from our initial analysis (see table S6), and after running the MCMC for 1000 generations (results in figure S6). This simulation will indicate whether we, using a time-dependent BiSSE model with arbitrarily defined time slices, may falsely infer imbalanced transition or extinction rates towards the tips of the trees, due to imbalance in tree shape, number of lineages and splitting events during these time slices (see table S2).

Third, we used the trait-dependent diversification process to simultaneously evolve 10 phylogenetic trees and a binary trait (with states A and B). We started the simulation at 37 Ma in order to obtain phylogenetic trees of approximately 1774 tips, similar to the empirical data. First, we simulated an extinction rate shift at 2.6 Ma for one of the trait states (extinction rate from 0.02 to 0.3), whereas the other trait state kept the same extinction rate (extinction rate = 0.02). The effect of the trait on speciation and transition rates remained constant between trait states and through time (speciation rate for both trait states = 0.2; transition rate for both trait states = 0.02). Similarly, we simulated a transition rate shift at 2.6 Ma for one of the trait states (transition rate from 0.005 to 0.34), whereas the other trait state kept the same transition rate (transition rate = 0.005). The effect of the trait on speciation and extinction rates remained constant between trait states and through time (speciation rate for both trait states = 0.2; extinction rate for both trait states = 0.02). We evaluated the changes in 95% Bayesian credible intervals in speciation, extinction and transition rates over time given the selected models from our initial analysis (see table S6), and after running the MCMC for 1000 generations (results in figure S7). These simulations were done to test whether we may correctly infer an increase in extinction or transition rates for one of the trait states in the Quaternary when it is truly there.

Sensitivity analyses

We performed two sensitivity analyses to assess the robustness of our results with respect to our classification of palms with megafaunal fruits. First, to evaluate whether our arbitrary cut-off value to classify palms with megafaunal fruits as those with fruits ≥ 4 cm affects the results, we repeated the time-dependent diversification analyses using cut-off values of fruits ≥ 3.5 cm and ≥ 4.5 cm to classify palm species with megafaunal fruits (n = 260 for 3.5 cm analysis; n = 186 for 4.5 cm analysis) versus small fruits (n = 1514 for 3.5 cm analysis; n = 1588 for 4.5 cm analysis). We evaluated the changes in 95% Bayesian credible intervals in extinction and transition rates over time given the selected models from our initial analysis (see table S6), and after running the MCMC for 10000 generations on 100 empirical palm phylogenetic trees (results in figure S10). Sampling fractions reflecting species and their traits (small or megafaunal fruits) sampled from the total were corrected for these datasets (33% of species with small fruits and 8% of species with megafaunal fruits were not sampled in the 3.5 cm dataset; 32% of species with small fruits and 6% of species with megafaunal fruits were not sampled in the 4.5 cm dataset).

To evaluate whether the inclusion of palms with large (≥ 4 cm), nutlike fruits that may be dispersed by rodents rather than megafauna influences our results, we excluded the subtribe Attaleinae in the Cocoseae from the data, and repeated the analyses. The Attaleinae typically includes palm species with large, nutlike fruits [25], and our Attaleinae sampling comprises 46 species with small fruits and 52 species with megafaunal fruits. We evaluated the changes in 95% Bayesian credible intervals in extinction and transition rates over time given the selected models from our initial analysis (see table S6), and after running the

MCMC for 2000 generations on 20 empirical palm phylogenetic trees (results in figure S11). Sampling fractions reflecting species and their traits (small or megafaunal fruits) sampled from the total were corrected for this dataset (33% of species with small fruits and 10% of species with megafaunal fruits were not sampled in this dataset).

Ancestral state reconstructions

To infer the ancestral fruit type (small or megafaunal) in palms, we explored two different methods to perform ancestral state reconstructions. First, we used stochastic character mapping to sample fruit size histories from their posterior probability distribution [26]. This was done by sampling 500 stochastic character maps using a Markov chain Monte Carlo on the palm Maximum Clade Credibility (MCC) phylogenetic tree under the unequal transition rate model (i.e. the transition rate from small to megafaunal fruits is different from the transition rate from megafaunal to small fruits). This resulted in a sample of unambiguous histories for small vs. megafaunal fruits over the phylogeny. We summarized these by showing the posterior probability that the edges and nodes of the tree are inferred to have megafaunal fruits (as compared to small fruits). Analyses were performed using the 'make.simmap' function in the 'phytools' R package [27]. Results are shown in figure 2.

Second, we used the parameters from the global BiSSE model (table S3) to reconstruct marginal ancestral states for small and megafaunal fruits on the palm MCC phylogenetic tree, using the asr.marginal function in the 'diversitree' R package [20].These reconstructions take the effect of fruit size on speciation and extinction rates into consideration, as ancestral states may not be independent from these rates. Results are shown in figure S4. Both methods provided qualitatively similar results.

References

1. Baker, W.J., et al., *Complete generic-level phylogenetic analyses of palms (Arecaceae) with comparisons of supertree and supermatrix approaches.* Systematic Biology, 2009. **58**: p. 240-256.
2. Faurby, S., et al., *An all-evidence species-level supertree for the palms (Arecaceae).* Molecular Phylogenetics and Evolution, 2016. **100**: p. 57-69.
3. Couvreur, T.L.P., F. Forest, and W.J. Baker, *Origin and global diversification patterns of tropical rain forests: inferences from a complete genus-level phylogeny of palms.* BMC Biology, 2011. **9**(1): p. 1-12.
4. Rambaut, A. and A. Drummond, *TreeAnnotator version 1.8.2 [computer program]* http://beast.bio.ed.ac.uk . 2015.
5. Janzen, D.H. and P.S. Martin, *Neotropical anachronisms: the fruits the gomphotheres ate.* Science, 1982. **215**(4528): p. 19-27.
6. Guimarães, P.R., Jr., M. Galetti, and P. Jordano, *Seed dispersal anachronisms: rethinking the fruits extinct megafauna ate.* PLoS ONE, 2008. **3**(3): p. e1745.
7. Bruggeman, J., J. Heringa, and B.W. Brandt, *PhyloPars: estimation of missing parameter values using phylogeny.* Nucleic Acids Research, 2009. **37**(Web Server issue): p. W179-W184.
8. Govaerts, R., et al., *World Checklist of Arecaceae*. 2015, Richmond (UK): Facilitated by the Royal Botanic Gardens, Kew. Published on the Internet; http://wcsp.science.kew.org. Retrieved June.
9. Kissling, W.D., et al., *Cenozoic imprints on the phylogenetic structure of palm species assemblages worldwide.* Proceedings of the National Academy of Sciences, 2012. **109**(19): p. 7379-7384.
10. Baker, W.J. and T.L.P. Couvreur, *Global biogeography and diversification of palms sheds light on the evolution of tropical lineages. II. Diversification history and origin of regional assemblages.* Journal of Biogeography, 2013. **40**(2): p. 286-298.
11. Barnosky, A.D., et al., *Assessing the causes of Late Pleistocene extinctions on the continents.* Science, 2004. **306**(5693): p. 70-75.
12. Weigelt, P., et al., *Late Quaternary climate change shapes island biodiversity.* Nature, 2016. **532**(7597): p. 99-102.
13. Kissling, W.D., K. Böhning–Gaese, and W. Jetz, *The global distribution of frugivory in birds.* Global Ecology and Biogeography, 2009. **18**(2): p. 150-162.
14. Kissling, W.D., et al., *Establishing macroecological trait datasets: digitalization, extrapolation, and validation of diet preferences in terrestrial mammals worldwide.* Ecology and Evolution, 2014. **4**(14): p. 2913-2930.
15. Dunning, J.B., *Handbook of avian body masses 2nd edn*. 2008, Boca Raton: CRC Press.
16. Faurby, S. and J.-C. Svenning, *Resurrection of the island rule: human-driven extinctions have obscured a basic evolutionary pattern.* American Naturalist, 2016. **187**(6): p. 812-820.
17. Faurby, S. and J.C. Svenning, *Historic and prehistoric human-driven extinctions have reshaped global mammal diversity patterns.* Diversity and Distributions, 2015. **21**(10): p. 1155-1166.
18. FitzJohn, R.G., W.P. Maddison, and S.P. Otto, *Estimating trait-dependent speciation and extinction rates from incompletely resolved phylogenies.* Systematic Biology, 2009. **58**(6): p. 595-611.

19. Maddison, W.P., P.E. Midford, and S.P. Otto, *Estimating a binary character's effect on speciation and extinction.* Systematic Biology, 2007. **56**(5): p. 701-710.
20. FitzJohn, R.G., *Diversitree: comparative phylogenetic analyses of diversification in R.* Methods in Ecology and Evolution, 2012. **3**(6): p. 1084-1092.
21. Rabosky, D.L. and E.E. Goldberg, *Model inadequacy and mistaken inferences of trait-dependent speciation.* Systematic Biology, 2015. **64**(2): p. 340-355.
22. Lewis, P.O., *A likelihood approach to estimating phylogeny from discrete morphological character data.* Systematic Biology, 2001. **50**: p. 913–925.
23. Höhna, S., M.R. May, and B.R. Moore, *TESS: an R package for efficiently simulating phylogenetic trees and performing Bayesian inference of lineage diversification rates.* Bioinformatics, 2016. **32**(5): p. 789-791.
24. May Michael, R., et al., *A Bayesian approach for detecting the impact of mass-extinction events on molecular phylogenies when rates of lineage diversification may vary.* Methods in Ecology and Evolution, 2016. **7**(8): p. 947-959.
25. Dransfield, J., et al., *Genera Palmarum: The evolution and classification of palms*. 2008, Kew, UK: Kew Publishing.
26. Huelsenbeck, J.P., R. Nielsen, and J.P. Bollback, *Stochastic mapping of morphological characters.* Syst Biol, 2003. **52**(2): p. 131-158.
27. Revell, L.J., *phytools: an R package for phylogenetic comparative biology (and other things).* Methods in Ecology and Evolution, 2012. **3**(2): p. 217-223.

Supplementary tables

Table S1: Biogeographic distribution of palm genera that contain species with small (< 4 cm in length) vs. megafaunal (≥ 4 cm in length) fruits. Columns summarize the mean, minimum (min) and maximum (max) fruit length in cm, the sample size (i.e. number of species), the percentage (%) of taxa with small vs. megafaunal fruits from the total in the region, and examples of palm genera with these typical fruit types (% of species in genus with these fruits, if not mentioned indicates 100%).

**Biogeographic region**	**Fruit type**	**Fruit length mean (min – max) [cm]**	**Sample size**	**%**	**Typical small- and megafaunal-fruited lineages (% of species with fruit type)**
Global	Small	1.55 (0.21–3.9)	1607	88%	*See NW and OW*
	Megafaunal	6.76 (4 – 35)	227	12%	*See NW and OW*
New World (NW)	Small	2.2 (0.21 – 3.9)	569	84%	*Acoelorrhaphe, Asterogyne, Barcella, Brahea, Butia, Calyptrogyne, Calyptronoma, Ceroxylon, Chamaedorea, Chelyocarpus, Colpothrinax, Copernicia, Cryosophila, Desmoncus, Dictyocaryum, Euterpe, Gaussia, Geonoma, Hemithrinax, Hyospathe, Iriartea, Iriartella, Itaya, Juania, Jubaea, Leopoldinia, Lepidocaryum, Leucothrinax, Lytocaryum, Neonicholsonia, Oenocarpus, Pholidostachys, Prestoea, Pseudophoenix, Reinhardtia, Rhapidophyllum, Roystonea, Sabal, Schippia, Serenoa, Synechanthus, Thrinax, Trithrinax, Washingtonia, Welfia, Wendlandiella, Zombia*
	Megafaunal	6.77 (4 – 35)	110	16%	*Ammandra, Aphandra, Manicaria, Mauritia, Pelagodoxa, Phytelephas, Acrocomia (33%), Aiphanes (9%), Allagoptera (20%), Astrocaryum (74%), Attalea (95%), Bactris (4%), Coccothrinax (5%), Mauritiella (33%), Parajubaea (67%), Pritchardia (37%), Socratea (25%), Syagrus (29%), Wettinia (20%)*
Old World (OW)	Small	1.55 (0.39– 3.8)	1038	90%	*Acanthophoenix, Actinokentia, Adonidia, Archontophoenix, Basselinia, Beccariophoenix, Bentinckia, Brassiophoenix, Carpentaria, Caryota, Ceratolobus, Chamaerops, Chuniophoenix, Clinosperma, Clinostigma, Cyphokentia, Cyphophoenix, Cyphosperma,*

					Cyrtostachys, Deckenia, Dictyosperma, Dransfieldia, Drymophloeus, Dypsis, Eleiodoxa, Eremospatha, Guihaia, Heterospathe, Hydriastele, Hyophorbe, Iguanura, Jubaeopsis, Kentiopsis, Korthalsia, Laccospadix, Laccosperma, Lanonia, Lemurophoenix, Lepidorrhachis, Linospadix, Livistona, Loxococcus, Marojejya, Masoala, Maxburretia, Myrialepis, Nephrosperma, Oncocalamus, Oncosperma, Oraniopsis, Phoenicophorium, Physokentia, Pigafetta, Plectocomia, Plectocomiopsis, Podococcus, Pogonotium, Ptychosperma, Retispatha, Rhopaloblaste, Rhopalostylis, Roscheria, Ravenea, Rhapis, Satakentia, Sommieria, Tahina, Tectiphiala, Trachycarpus, Verschaffeltia, Wallichia
	Megafaunal	6.71<br>(4 – 30)	117	10%	*Actinorhytis, Bismarckia, Borassodendron, Borassus, Carpoxylon, Eugeissona, Hedyscepe, Hyphaene, Kerriodoxa, Latania, Medemia, Metroxylon, Neoveitchia, Normanbya, Pholidocarpus, Raphia, Satranala, Voanioala, Wodyetia, Areca (31%), Arenga (25%), Balaka (20%), Burretiokentia (20%), Calamus (1%), Calyptrocalyx (4%), Chambeyronia (50%), Corypha (75%), Daemonorops (1%), Howea (50%), Johannesteijsmannia (50%), Licuala (1%), Nenga (40%), Orania (50%), Phoenix (7%), Pinanga (1%), Ponapea (33%), Ptychococcus (50%), Salacca (75%), Saribus (11%), Sclerosperma (33%), Veitchia (14%)*

Table S2: Summary of number of speciation events, number of lineages, transition rates and number of transitions for each time slice in this study. These are inferred from the palm phylogenetic data using the time-dependent BiSSE model. The number of transitions was calculated by multiplying the transition rate (in lineages / myr) for each of the states (small vs. megafaunal) by the number of lineages and the total time spend in each time slice.

**Time (Ma)**	**Speciation events**		**Lineages**	**Inferred transition rates (lineages / myr)**		**Transitions**	
	Towards present	Towards past	Total	Small to megafaunal	Megafaunal to small	Small to megafaunal	Megafaunal to small
0.5	35	1523	1774	0.035	0.721	31	640
1	144	1414	1690	0.021	0.338	35	571
2.6	458	1100	1611	0.011	0.122	48	511
5	805	753	1318	0.009	0.063	59	417
10	1192	366	925	0.008	0.040	70	367
15	1340	218	459	0.008	0.032	49	222
20	1411	147	282	0.007	0.029	39	162
25	1461	97	179	0.007	0.027	30	120

Table S3: Trait-dependent vs. time-dependent diversification model selection for global palms (BiSSE). Nine BiSSE models were fitted to the palm Maximum Clade Credibility (MCC) tree: eight trait-dependent diversification models, and one time- and trait-dependent diversification model. The best-fitting model within the 'trait-dependent diversification' models given the fewest number of parameters (i.e. 5 Df) is indicated in bold (*). This model indicates that small- and megafaunal-fruited palm lineages evolved with equal extinction rates. However, the full time-dependent (and trait-dependent) diversification model (12 Df) had the best fit to the data, suggesting that global palms show a diversification rate shift at the Neogene-Quaternary boundary.

	**Model constraints**	**Df**	**LnLik**	**AIC**	**ChiSq**	**P**
Trait-dependent diversification	Full (no constrain)	6	-5978.8	11970		
	$\lambda_{small} \sim \lambda_{megafaunal}$	5	-5990.1	11990	22.610	2e-06 ***
	*** $\mu_{small} \sim \mu_{megafaunal}$**	**5**	**-5979.4**	**11969**	**1.144**	**0.285**
	$q_{small \rightarrow megafaunal} \sim q_{megafaunal \rightarrow small}$	5	-5986.1	11982	14.590	0.0001 ***
	$\lambda_{small} \sim \lambda_{megafaunal}$, $\mu_{small} \sim \mu_{megafaunal}$	4	-6026.9	12062	96.045	< 2.2e-16 ***
	$\lambda_{small} \sim \lambda_{megafaunal}$, $q_{small \rightarrow megafaunal} \sim q_{megafaunal \rightarrow small}$	4	-5990.7	11989	23.621	7.4e-06 ***
	$\mu_{small} \sim \mu_{megafaunal}$, $q_{small \rightarrow megafaunal} \sim q_{megafaunal \rightarrow small}$	4	-5988.7	11985	19.732	5.2e-05 ***
	$\lambda_{small} \sim \lambda_{megafaunal}$, $\mu_{small} \sim \mu_{megafaunal}$, $q_{small \rightarrow megafaunal} \sim q_{megafaunal \rightarrow small}$	3	-6033.1	12072	108.491	< 2.2e-16 ***
Time- and trait-dependent diversification	*** Full (no constraint)**	**12**	**-5940.7**	**11906**		2.2e-14 ***

Df= degrees of freedom, LnLik = log likelihood, AIC = Akaike Information Criterion, ChiSq = Chi-square, P = significance of the model compared to the full model, λ = speciation rate, μ = extinction rate, q = transition rate, ~ = equal to (constrain). Significance codes: 0 '***' 0.001 '**' 0.01 '*' 0.05 '.' 0.1 ' ' 1

Table S4: Trait-dependent vs. time-dependent diversification model selection for New World palms (BiSSE). Two BiSSE models were fitted to the New World palm Maximum Clade Credibility (MCC) tree. The full time- and trait-dependent diversification model (12 Df) is indicated in bold (*). This model fitted the data significantly better than a model without time-dependent trait diversification. This suggests that New World palms show a diversification rate shift at the Neogene-Quaternary boundary.

	**Model constraints**	**Df**	**LnLik**	**AIC**	**ChiSq**	**P**
Trait-dependent diversification	Full (no time-dependent)	6	-2218.3	4448.6	60.043	4.4e-11 ***
Time- and trait-dependent diversification	*** Full (no constraint)**	**12**	**-2188.3**	**4400.6**		

Df= degrees of freedom, LnLik = log likelihood, AIC = Akaike Information Criterion, ChiSq = Chi-square, P = significance of the model compared to the full model, λ = speciation rate, μ = extinction rate, q = transition rate, ~ = equal to (constrain). Significance codes: 0 '***' 0.001 '**' 0.01 '*' 0.05 '.' 0.1 ' ' 1

Table S5: Trait-dependent vs. time-dependent diversification model selection for Old World palms (BiSSE). Two BiSSE models were fitted to the Old World palm Maximum Clade Credibility (MCC) tree. The full time- and trait-dependent diversification model (12 Df) is indicated in bold (*). This model fitted the data significantly better than a model without time-dependent trait diversification. This suggests that Old World palms show a diversification rate shift at the Neogene-Quaternary boundary.

	**Model constraints**	**Df**	**LnLik**	**AIC**	**ChiSq**	**P**
Trait-dependent diversification	Full (no time-dependent)	6	-3754.9	7521.8	70.192	3.7e-13 ***
Time- and trait-dependent diversification	*** Full (no constraint)**	**12**	**-3719.8**	**7463.6**		

Df= degrees of freedom, LnLik = log likelihood, AIC = Akaike Information Criterion, ChiSq = Chi-square, P = significance of the model compared to the full model, λ = speciation rate, μ = extinction rate, q = transition rate, ~ = equal to (constrain). Significance codes: 0 '***' 0.001 '**' 0.01 '*' 0.05 '.' 0.1 ' ' 1

Table S6: Time-dependent model selection for global palms (BiSSE). We used a step-wise approach to select the best time-dependent diversification model given the global palm dataset, starting with the most complex model (the full time- and trait-dependent diversification model) and applying parameter constraints to evaluate the fit of simpler models using likelihood-ratio tests (nested models) and the Akaike Information Criterion (AIC, non-nested models). These constraints could be trait dependent (e.g. speciation rates between small- and megafaunal-fruited palm lineages are constrained to be similar) or time-dependent (e.g. pre-Quaternary megafaunal lineages and Quaternary megafaunal lineages are constrained to be similar), or a combination of these. For global palms, 45 time-dependent BiSSE models were fitted to the palm Maximum Clade Credibility (MCC) tree. The best-fitting initial models given the fewest number of parameters (i.e. 10 Df) are indicated in bold. After the initial model selection the best models were combined into even simpler (less parameter-rich) models ('extra models tested'). These simpler models, however, were rejected. The best model (with the lowest AIC) is indicated in bold (*). In this model pre-Quaternary extinction rates of small- and megafaunal-fruited palm lineages are similar, as well as the pre-Quaternary transition rates from small to megafaunal fruits and vice versa. Letters (a-l) refer to rates illustrated in figure S8.

	**Model parameters (figure S8)**	**Df**	**LnLik**	**AIC**	**ChiSq**	**P**
	Full time- and trait-dependent model (no constraint)	12	-5940.7	11906		
**Constrain by time and trait**	All λ: $\lambda_a \sim \lambda_b \sim \lambda_c \sim \lambda_d$	9	-5958.6	11935	35.751	8.5e-08 ***
	All μ: $\mu_e \sim \mu_f \sim \mu_g \sim \mu_h$	9	-5953.8	11926	26.092	9.1e-06 ***
	All q: $q_i \sim q_j \sim q_k \sim q_l$	9	-5954.0	11926	26.426	7.8e-06 ***
	All λ and μ	6	-6017.9	12048	154.317	< 2.2e-16 ***
	All λ and q	6	-5971.3	11955	61.145	2.6e-11 ***
	All μ and q	6	-5974.9	11962	68.385	8.8e-13 ***
	All λ, μ and q	3	-6033.1	12072	184.683	< 2.2e-16 ***
**Constrain by time**	**$\lambda_a \sim \lambda_b, \lambda_c \sim \lambda_d$**	**10**	**-5943.6**	**11907**	**5.701**	**0.058 .**
	$\mu_e \sim \mu_f, \mu_g \sim \mu_h$	10	-5944.1	11908	6.752	0.034*
	$q_i \sim q_j, q_k \sim q_l$	10	-5947.8	11916	14.126	0.001 ***
**Constrain by trait**	$\lambda_a \sim \lambda_c, \lambda_b \sim \lambda_d$	10	-5952.7	11925	23.960	6.3e-06 ***
	$\mu_e \sim \mu_g, \mu_f \sim \mu_h$	10	-5952.2	11924	22.841	1.1e-05

						***
	$q_i \sim q_j$, $q_k \sim q_l$	10	-5954.0	11928	26.431	1.8e-06 ***
**Constrain in pre-Quaternary**	$\lambda_a \sim \lambda_b$	11	-5942.0	11906	2.457	0.117
	$\mu_e \sim \mu_f$	11	-5941.3	11904	1.033	0.31
	$q_i \sim q_j$	11	-5941.6	11905	1.625	0.202
	$\lambda_a \sim \lambda_b$, $\mu_e \sim \mu_f$	10	-5954.8	11930	28.209	7.5e-07 ***
	**$\lambda_a \sim \lambda_b$, $q_i \sim q_j$**	**10**	**-5942.0**	**11904**	**2.592**	**0.274**
	*** $\mu_e \sim \mu_f$, $q_i \sim q_j$**	**10**	**-5941.6**	**11903**	**1.739**	**0.419**
	$\lambda_a \sim \lambda_b$, $\mu_e \sim \mu_f$, $q_i \sim q_j$	9	-5956.4	11931	31.280	7.4e-07 ***
**Constrain in Quaternary**	$\lambda_c \sim \lambda_d$	11	-5943.7	11910	5.986	0.014 *
	$\mu_g \sim \mu_h$	11	-5940.7	11904	0.006	0.938
	$q_k \sim q_l$	11	-5948.1	11918	14.744	0.0001 ***
	$\lambda_c \sim \lambda_d$, $\mu_g \sim \mu_h$	10	-5950.1	11920	18.637	9e-05 ***
	$\lambda_c \sim \lambda_d$, $q_k \sim q_l$	10	-5948.1	11916	14.661	0.001 ***
	$\mu_g \sim \mu_h$, $q_k \sim q_l$	10	-5950.6	11921	19.768	5.1e-05 ***
	$\lambda_c \sim \lambda_d$, $\mu_g \sim \mu_h$, $q_k \sim q_l$	9	-5959.6	11937	37.769	3.2e-08 ***
**Constrain by small fruits**	$\lambda_a \sim \lambda_c$	11	-5952.1	11926	22.626	2e-06 ***
	$\mu_e \sim \mu_g$	11	-5952.0	11926	22.460	2.1e-06 ***
	$q_i \sim q_k$	11	-5943.7	11909	5.900	0.015 *
	$\lambda_a \sim \lambda_c$, $\mu_e \sim \mu_g$	10	-5961.3	11943	41.071	1.2e-09 ***
	$\lambda_a \sim \lambda_c$, $q_i \sim q_k$	10	-5954.4	11929	27.353	1.1e-06 ***
	$\mu_e \sim \mu_g$, $q_i \sim q_k$	10	-5954.7	11929	27.942	8.6e-07 ***
	$\lambda_a \sim \lambda_c$, $\mu_e \sim \mu_g$, $q_i \sim q_k$	9	-5962.9	11944	44.405	1.2e-09 ***
**Constrain by megafaunal fruits**	$\lambda_b \sim \lambda_d$	11	-5941.4	11905	1.263	0.261
	$\mu_f \sim \mu_h$	11	-5940.9	11904	0.255	0.614
	$q_j \sim q_l$	11	-5947.6	11917	13.718	0.0002 ***
	**$\lambda_b \sim \lambda_d$, $\mu_f \sim \mu_h$**	**10**	**-5941.8**	**11904**	**2.199**	**0.333**
	$\lambda_b \sim \lambda_d$, $q_j \sim q_l$	10	-5948.0	11916	14.457	0.0001***
	$\mu_f \sim \mu_h$, $q_j \sim q_l$	10	-5948.0	11916	14.475	0.001 ***

	$\lambda_b \sim \lambda_d, \mu_f \sim \mu_h, q_j \sim q_l$	9	-5948.5	11915	15.413	0.001 **
**Extra, simpler models tested**	$\lambda_b \sim \lambda_d, \mu_f \sim \mu_h, q_i \sim q_k$	9	-5944.7	11907	7.8603	0.049 *
	$\lambda_a \sim \lambda_b, \lambda_c \sim \lambda_d, q_i \sim q_k$	9	-5945.1	11908	8.6178	0.035 *

Df= degrees of freedom, LnLik = log likelihood, AIC = Akaike Information Criterion, ChiSq = Chi-square, P = significance of the model compared to the full model, λ = speciation rate, μ = extinction rate, q = transition rate. Significance codes: 0 '***' 0.001 '**' 0.01 '*' 0.05 '.' 0.1 ' ' 1

Table S7: Time-dependent model selection for New World palms (BiSSE). We used a step-wise approach to select the best time-dependent diversification model given the New World palm dataset, starting with the most complex model (the full time- and trait-dependent diversification model) and applying parameter constraints to evaluate the fit of simpler models using likelihood-ratio tests (nested models) and Akaike Information Criterion (AIC, non-nested models). These constraints could be trait dependent (e.g. speciation rates between small- and megafaunal-fruited palm lineages are constrained to be similar) or time-dependent (e.g. pre-Quaternary megafaunal lineages and Quaternary megafaunal lineages are constrained to be similar), or a combination of these. For New World palms, 24 time-dependent BiSSE models were fitted to the palm Maximum Clade Credibility (MCC) tree. From the initial models, the model in which all transition rates were constrained to be equal was not rejected compared to the full model. All subsequent models, testing variations on speciation and extinction rate constraints, therefore included the transition rate constrain. The best-fitting model given the fewest number of parameters (i.e. 8 Df) is indicated in bold (*). In this model all transition rates are equal, as well as speciation rates of small- and megafaunal-fruited palm lineages in the Quaternary. Letters (a-l) refer to rates illustrated in figure S8.

	**Model parameters (figure S8)**	**Df**	**LnLik**	**AIC**	**ChiSq**	**P**
	Full time- and trait-dependent model (no constraint)	12	-2188.3	4400.6		
**Constrain by time and trait**	All λ: $\lambda_a \sim \lambda_b \sim \lambda_c \sim \lambda_d$	9	-2197.1	4412.1	17.550	0.0005 ***
	All μ: $\mu_e \sim \mu_f \sim \mu_g \sim \mu_h$	9	-2215.6	4449.2	54.601	8.4e-12 ***
	**All q: $q_i \sim q_j \sim q_k \sim q_l$**	**9**	**-2190.9**	**4399.9**	**5.326**	**0.149**
	All λ and μ	6	-2219.4	4450.8	62.228	1.6e-11 ***
	All λ and q	6	-2206.9	4425.8	37.236	1.6e-06 ***
	All μ and q	6	-2225.8	4463.6	75.074	3.7e-14 ***
	All λ, μ and q	3	-2233.3	4472.7	90.106	1.6e-15 ***
**Constrain by time**	$q_i \sim q_j \sim q_k \sim q_l$, $\lambda_a \sim \lambda_b$, $\lambda_c \sim \lambda_d$	7	-2198.1	4410.1	19.554	0.002 **
	$q_i \sim q_j \sim q_k \sim q_l$, $\mu_e \sim \mu_f$, $\mu_g \sim \mu_h$	7	-2199.9	4413.7	23.158	0.0003 ***
**Constrain by trait**	$q_i \sim q_j \sim q_k \sim q_l$, $\lambda_a \sim \lambda_c$, $\lambda_b \sim \lambda_d$	7	-2205.3	4424.6	34.090	2.3e-06 ***
	$q_i \sim q_j \sim q_k \sim q_l$, $\mu_e \sim \mu_g$, $\mu_f \sim \mu_h$	7	-2220.9	4455.8	65.213	1e-12 ***

**Constrain in pre-Quaternary**	$q_i \sim q_j \sim q_k \sim q_l$, $\lambda_a \sim \lambda_b$	8	-2198.1	4412.1	19.553	0.0006***
	$q_i \sim q_j \sim q_k \sim q_l$, $\mu_e \sim \mu_f$	8	-2199.1	4414.2	21.623	0.0002382 ***
	$q_i \sim q_j \sim q_k \sim q_l$, $\lambda_a \sim \lambda_b$, $\mu_e \sim \mu_f$	7	-2200.6	4415.2	24.656	0.0002 ***
**Constrain in Quaternary**	***$q_i \sim q_j \sim q_k \sim q_l$, $\lambda_c \sim \lambda_d$**	**8**	**-2192.6**	**4401.1**	**8.537**	**0.074 .**
	$q_i \sim q_j \sim q_k \sim q_l$, $\mu_g \sim \mu_h$	8	-2198.8	4413.5	20.964	0.0003 ***
	$q_i \sim q_j \sim q_k \sim q_l$, $\lambda_c \sim \lambda_d$, $\mu_g \sim \mu_h$	7	-2199.9	4413.8	23.234	0.0003045 ***
**Constrain by small fruits**	$q_i \sim q_j \sim q_k \sim q_l$, $\lambda_a \sim \lambda_c$	8	-2197.6	4411.1	18.568	0.001 ***
	$q_i \sim q_j \sim q_k \sim q_l$, $\mu_e \sim \mu_g$	8	-2217.0	4450.1	57.522	9.6e-12 ***
	$q_i \sim q_j \sim q_k \sim q_l$, $\lambda_a \sim \lambda_c$, $\mu_e \sim \mu_g$	7	-2216.6	4447.2	56.630	6e-11 ***
**Constrain by large fruits**	$q_i \sim q_j \sim q_k \sim q_l$, $\lambda_b \sim \lambda_d$	8	-2200.3	4416.6	24.084	7.7e-05 ***
	$q_i \sim q_j \sim q_k \sim q_l$, $\mu_f \sim \mu_h$	8	-2195.8	4407.7	15.114	0.004**
	$q_i \sim q_j \sim q_k \sim q_l$, $\lambda_b \sim \lambda_d$, $\mu_f \sim \mu_h$	7	-2200.4	4414.8	24.229	0.0002 ***

Df= degrees of freedom, LnLik = log likelihood, AIC = Akaike Information Criterion, ChiSq = Chi-square, P = significance of the model compared to the full model, λ = speciation rate, μ = extinction rate, q = transition rate. Significance codes: 0 '***' 0.001 '**' 0.01 '*' 0.05 '.' 0.1 ' ' 1

Table S8: Time-dependent model selection for Old World palms (BiSSE). We used a step-wise approach to select the best time-dependent diversification model given the Old World palm dataset, starting with the most complex model (the full time- and trait-dependent diversification model) and applying parameter constraints to evaluate the fit of simpler models using likelihood-ratio tests (nested models) and Akaike Information Criterion (AIC, non-nested models). These constraints could be trait dependent (e.g. speciation rates between small- and megafaunal-fruited palm lineages are constrained to be similar) or time-dependent (e.g. pre-Quaternary megafaunal lineages and Quaternary megafaunal lineages are constrained to be similar), or a combination of these. For Old World palms, 47 time-dependent BiSSE models were fitted to the palm Maximum Clade Credibility (MCC) tree. The best-fitting initial models given the fewest number of parameters (i.e. 8, 9 or 10 Df) are indicated in bold. After initial model selection the best models were combined into even simpler (less parameter-rich) models ('extra models tested'). In the Old World, these simpler models were not rejected; the best models (with the lowest AIC) are indicated with *. These models indicate that pre-Quaternary and Quaternary speciation rates of small-fruited lineages, and pre-Quaternary and Quaternary speciation and extinction rates of megafaunal-fruited lineages, have been similar, and that the transition rate from small to megafaunal fruits has been constant through time as well. Letters (a-l) refer to rates illustrated in figure S8.

	**Model parameters (figure S8)**	**Df**	**LnLik**	**AIC**	**ChiSq**	**P**
	Full time- and trait-dependent model (no constraint)	12	-3719.8	7463.6		
**Constrain by time and trait**	All λ: $\lambda_a \sim \lambda_b \sim \lambda_c \sim \lambda_d$	9	-3726.9	7471.7	14.124	0.003**
	All μ: $\mu_e \sim \mu_f \sim \mu_g \sim \mu_h$	9	-3732.1	7482.1	24.481	2e-05 ***
	All q: $q_i \sim q_j \sim q_k \sim q_l$	9	-3743.6	7505.2	47.573	2.6e-10 ***
	All λ and μ	6	-3816.0	7644.0	192.349	< 2.2e-16 ***
	All λ and q	6	-3760.4	7532.9	81.239	2e-15 ***
	All μ and q	6	-3760.4	7532.9	81.239	2e-15 ***
	All λ, μ and q	3	-3823.0	7652.1	206.471	<2.2e-16 ***
**Constrain by time**	$\lambda_a \sim \lambda_b, \lambda_c \sim \lambda_d$	10	-3724.4	7468.7	9.106	0.011 *
	$\mu_e \sim \mu_f, \mu_g \sim \mu_h$	10	-3729.6	7479.2	19.576	5.613e-05 ***
	$q_i \sim q_j, q_k \sim q_l$	10	-3731.7	7483.3	23.677	7.2e-06 ***
**Constrain by trait**	**$\lambda_a \sim \lambda_c, \lambda_b \sim \lambda_d$**	**10**	**-3721.5**	**7463.0**	**3.412**	**0.182**
	$\mu_e \sim \mu_g, \mu_f \sim \mu_h$	10	-3725.4	7470.7	11.123	0.004 **
	$q_i \sim q_j, q_k \sim q_l$	10	-3742.6	7505.1	45.509	1.3e-10

						***
**Constrain in pre-Quaternary**	$\lambda_a \sim \lambda_b$	11	-3722.8	7467.6	6.013	0.014 *
	$\mu_e \sim \mu_f$	11	-3721.6	7465.2	3.540	0.06 .
	$q_i \sim q_j$	11	-3720.2	7462.4	0.820	0.365
	$\lambda_a \sim \lambda_b, \mu_e \sim \mu_f$	10	-3741.5	7503.1	43.473	3.6e-10 ***
	$\lambda_a \sim \lambda_b, q_i \sim q_j$	10	-3730.2	7480.3	20.697	3.2e-05 ***
	$\mu_e \sim \mu_f, q_i \sim q_j$	10	-3727.8	7475.7	16.051	0.0003***
	$\lambda_a \sim \lambda_b, \mu_e \sim \mu_f, q_i \sim q_j$	9	-3748.4	7514.8	57.157	2.4e-12 ***
**Constrain in Quaternary**	$\lambda_c \sim \lambda_d$	11	-3724.5	7471.0	9.354	0.002 **
	$\mu_g \sim \mu_h$	11	-3719.8	7461.7	0.027	0.87
	$q_k \sim q_l$	11	-3726.4	7474.7	13.117	0.0003 ***
	$\lambda_c \sim \lambda_d, \mu_g \sim \mu_h$	10	-3736.4	7492.7	33.111	6.5e-08 ***
	$\lambda_c \sim \lambda_d, q_k \sim q_l$	10	-3726.6	7473.2	13.622	0.001 **
	$\mu_g \sim \mu_h, q_k \sim q_l$	10	-3737.8	7495.6	35.957	1.6e-08 ***
	$\lambda_c \sim \lambda_d, \mu_g \sim \mu_h, q_k \sim q_l$	9	-3751.4	7520.9	63.237	1.2e-13 ***
**Constrain by small fruits**	$\lambda_a \sim \lambda_c$	11	-3721.5	7465.0	3.335	0.068 .
	$\mu_e \sim \mu_g$	11	-3725.3	7472.7	11.052	0.0009 ***
	$q_i \sim q_k$	11	-3720.1	7462.1	0.501	0.479
	$\lambda_a \sim \lambda_c, \mu_e \sim \mu_g$	10	-3726.3	7472.6	12.958	0.002 **
	**$\lambda_a \sim \lambda_c, q_i \sim q_k$**	**10**	**-3721.8**	**7463.6**	**3.978**	**0.137**
	$\mu_e \sim \mu_g, q_i \sim q_k$	10	-3725.8	7471.6	11.958	0.003 **
	$\lambda_a \sim \lambda_c, \mu_e \sim \mu_g, q_i \sim q_k$	9	-3726.7	7471.3	13.694	0.003 **
**Constrain by large fruits**	$\lambda_b \sim \lambda_d$	11	-3719.9	7461.7	0.105	0.745
	$\mu_f \sim \mu_h$	11	-3719.8	7461.7	0.042	0.838
	$q_j \sim q_l$	11	-3737.1	7496.2	34.562	4.1e-09 ***
	**$\lambda_b \sim \lambda_d, \mu_f \sim \mu_h$**	**10**	**-3719.9**	**7459.8**	**0.134**	**0.935**
	$\lambda_b \sim \lambda_d, q_j \sim q_l$	10	-3737.4	7494.8	35.148	2.3e-08 ***
	$\mu_f \sim \mu_h, q_j \sim q_l$	10	-3737.1	7494.2	34.579	3.1e-08 ***
	$\lambda_b \sim \lambda_d, \mu_f \sim \mu_h, q_j \sim q_l$	9	-3737.4	7492.8	35.169	1.1e-07 ***
**Extra models tested**	*** $\lambda_a \sim \lambda_c, \lambda_b \sim \lambda_d, \mu_f \sim \mu_h, q_i \sim q_k$**	**8**	**-3721.9**	**7459.7**	**4.0879**	**0.394**
	$\lambda_a \sim \lambda_c, \lambda_b \sim \lambda_d, q_i \sim q_k$	9	-3721.9	7461.7	4.0918	0.252

$\lambda_a \sim \lambda_c$, $\lambda_b \sim \lambda_d$, $\mu_f \sim \mu_{h,}$	9	-3721.5	7461.1	3.4631	0.326
$\lambda_a \sim \lambda_c$, $q_i \sim q_k$, $\mu_f \sim \mu_{h,}$	9	-3721.8	7461.6	3.9990	0.262
*** $\lambda_b \sim \lambda_d$, $\mu_f \sim \mu_h$, $q_i \sim q_k$**	**9**	**-3720.1**	**7458.3**	**0.6558**	**0.884**

Df= degrees of freedom, LnLik = log likelihood, AIC = Akaike Information Criterion, ChiSq = Chi-square, P = significance of the model compared to the full model, λ = speciation rate, μ = extinction rate, q = transition rate. Significance codes: 0 '***' 0.001 '**' 0.01 '*' 0.05 '.' 0.1 ' ' 1

Supplementary figures

Figure S1: Simulations of neutral binary traits (with state A and state B) on 10 random empirical palm phylogenies and the resulting speciation, extinction, net diversification and transition rates through time under several transition rate (q) parameters: q = 0.01 (a, b, c, d), q = 0.1 (e, f, g, h), q = 1 (i, j, k, l) and q = 10 (m, n, o, p). These transition rates reflect changes from state A to B and vice versa from rare (q = 0.01) to frequent (q = 10) changes. State A and B are expected to be neutral with respect to speciation, extinction, transition and net diversification rates. Results indicate that when traits are simulated under low transition rate parameters, extinction rates may show an (unexpected) increase towards the present (b and f), whereas this increase is less common under high transition rate parameters (j and n). Transition rates, however, do not show such an increase under any of the scenarios (d, h, l, p) but reflect the simulated scenario values (q = 0.01, 0.01, 1 or 10) respectively, as expected. Speciation rates also do not show a biased trend through time under any of the transition rate scenarios (a, e, i, m).

Figure S1

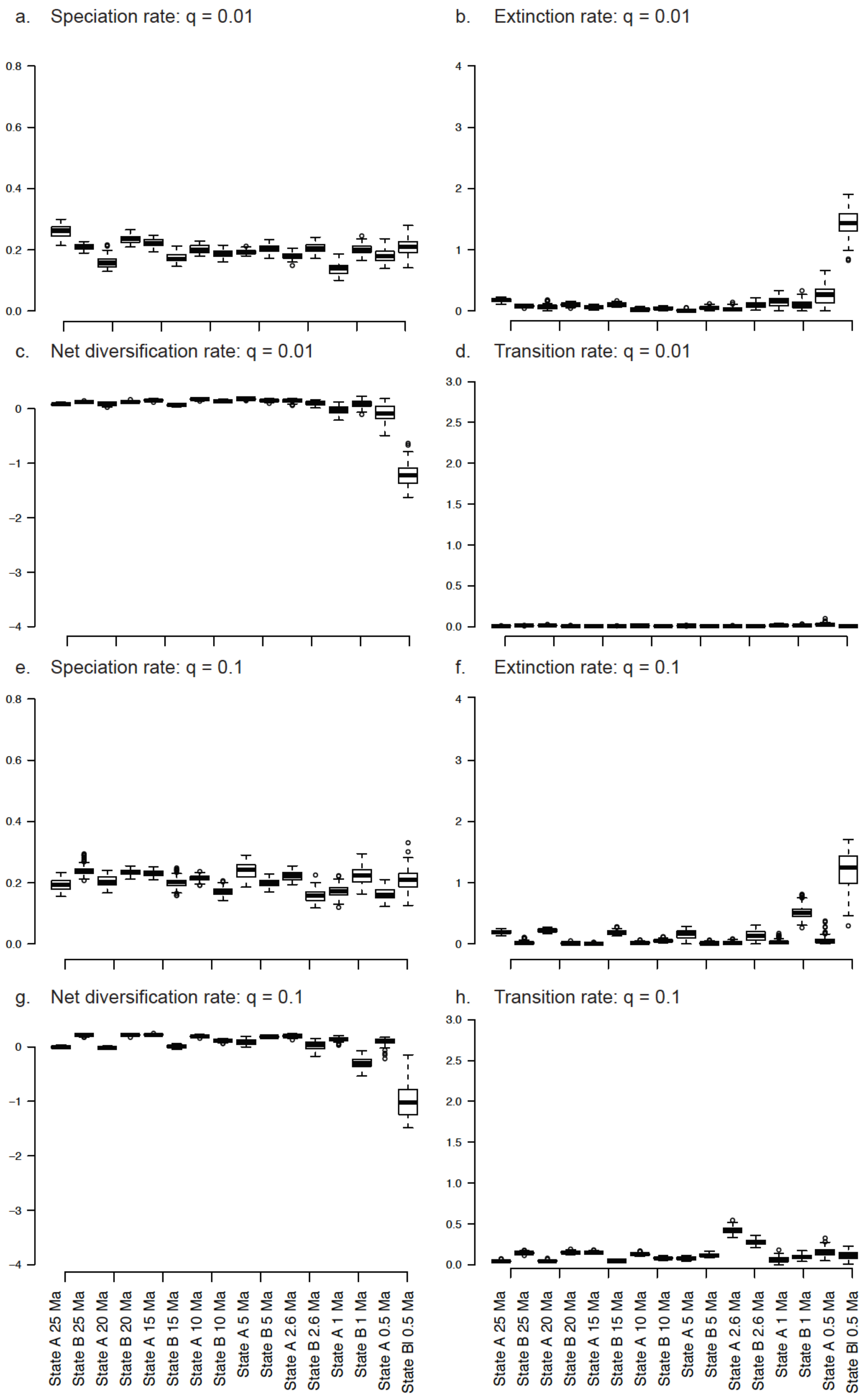

a. Speciation rate: q = 0.01
b. Extinction rate: q = 0.01
c. Net diversification rate: q = 0.01
d. Transition rate: q = 0.01
e. Speciation rate: q = 0.1
f. Extinction rate: q = 0.1
g. Net diversification rate: q = 0.1
h. Transition rate: q = 0.1
0.0
0.2
0.4
0.6
0.8
0
1
2
3
4
−1
−2
−3
−4
0.5
1.0
1.5
2.0
2.5
3.0
State A 25 Ma
State B 25 Ma
State A 20 Ma
State B 20 Ma
State A 15 Ma
State B 15 Ma
State A 10 Ma
State B 10 Ma
State A 5 Ma
State B 5 Ma
State A 2.6 Ma
State B 2.6 Ma
State A 1 Ma
State B 1 Ma
State A 0.5 Ma
State BI 0.5 Ma

Figure S1 (continued)

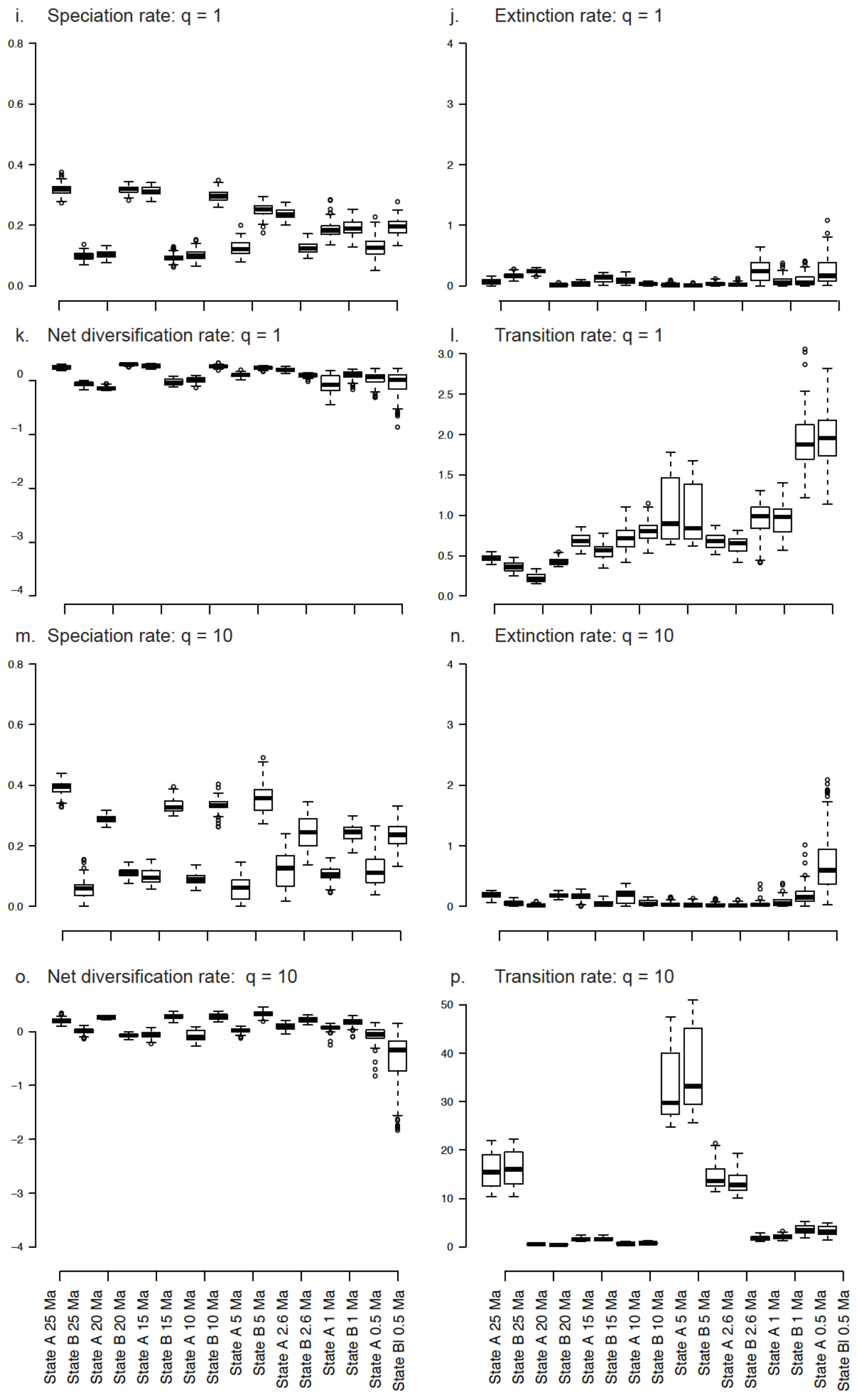

Figure S2: Simulations of neutral binary traits (with state A and state B) on 100 random empirical palm phylogenies and the resulting speciation (a), extinction (b), net diversification (c) and transition rate (d) through time when traits are simulated under the observed transition rate (q) estimates: $q_{megafaunal\ to\ small} = 0.017$; $q_{small\ to\ megafaunal} = 0.006$. These transition rates reflect changes from state A (hypothetical megafaunal fruits) to state B (hypothetical small fruits) and vice versa. State A and B are expected to be neutral with respect to speciation, extinction, transition and net diversification rates. Results indicate that when traits are simulated under the observed (empirical) transition rates, the extinction rate shows a moderate (unexpected) increase towards the present (b). The transition rate, however, does not show such an increase (d). The speciation rate also does not show a biased pattern through time (a).

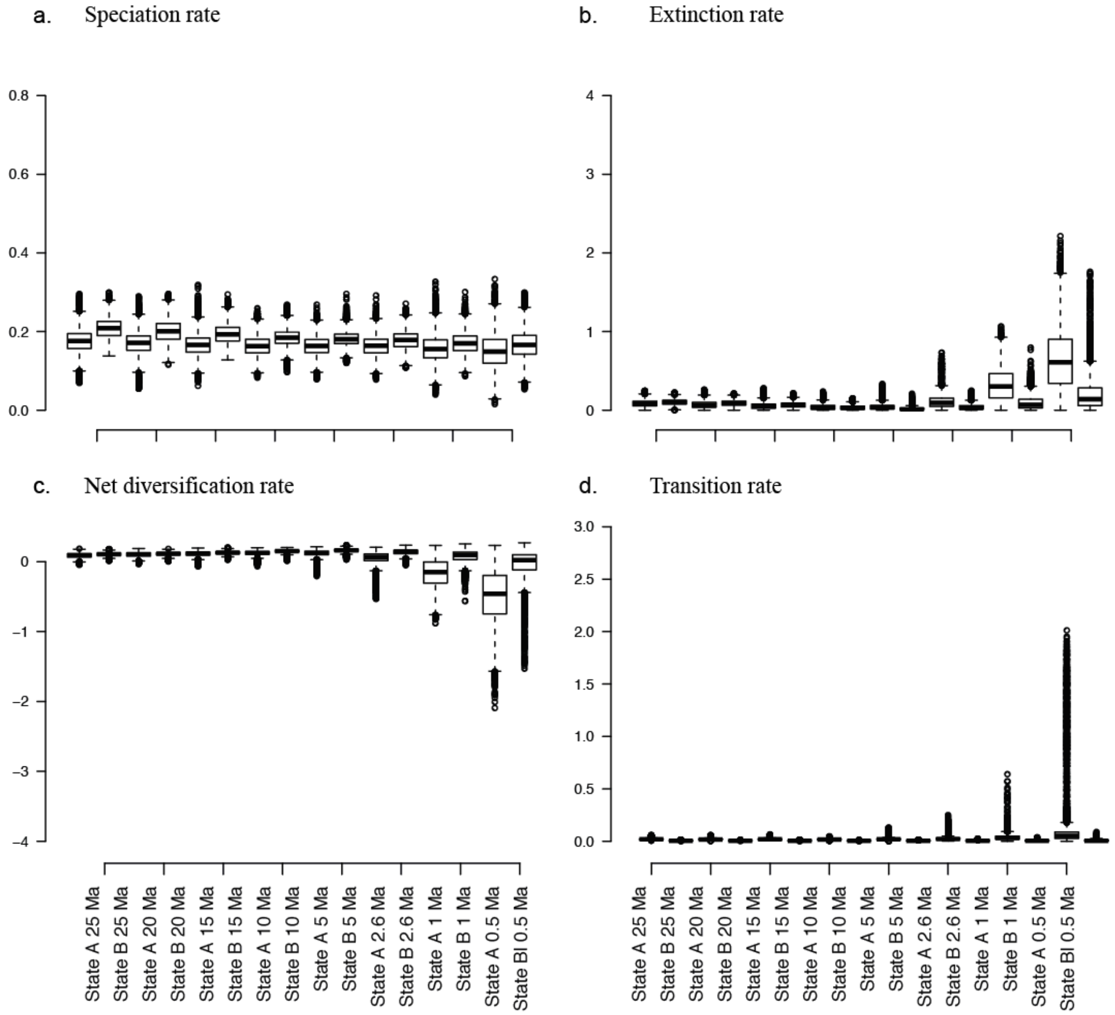

Figure S3: The relationship between fruit length and fruit diameter in palms (*n* = 1427). (a) Linear relationship between log(fruit length) and log(fruit diameter), in which dots represent species. The coefficients of the linear regression line are indicated. (b) Histogram of the ratio between fruit length and fruit diameter, showing that the majority of palm species have globular (roundish) fruits, in which fruit length and fruit diameter are similar.

a.

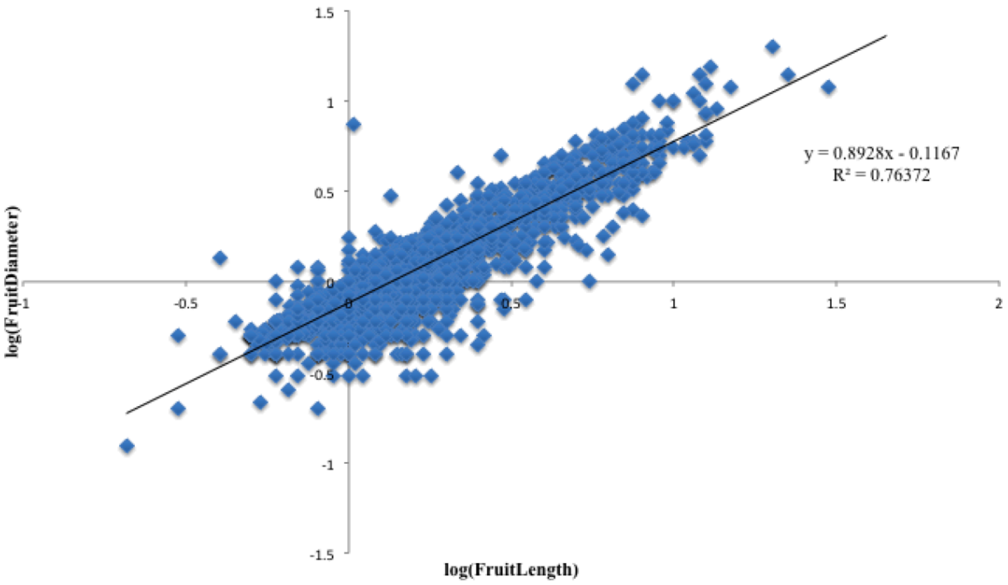

b.

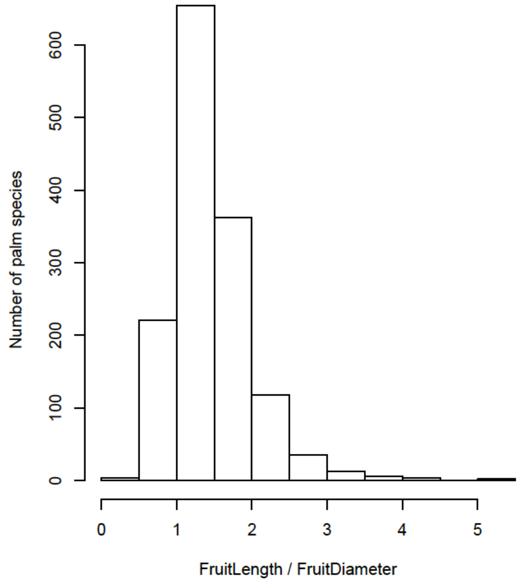

Figure S4: Macroevolution of megafaunal palm fruits (Arecaceae). The palm phylogeny shows the probability of megafaunal fruits (yellow) at the internal nodes on the Maximum Clade Credibility (MCC) tree of palms. The probabilities were derived from ancestral state reconstructions under an equal extinction-rate model (table S3). The reconstruction suggests that all palm fruits have evolved from the ancestral state of a megafaunal palm fruit (ca. 110 Ma). The five subfamilies are indicated at the branches of the tree. All palm genera that comprise at least one species with megafaunal fruits (*n* = 59) are indicated at the tips (colors of the names do not have a meaning). The evolution of small fruits from the megafaunal-fruited palm ancestor happened at least eleven times. Megafaunal fruits ≥ 4 cm length. All other palms with small fruits < 4 cm in length.

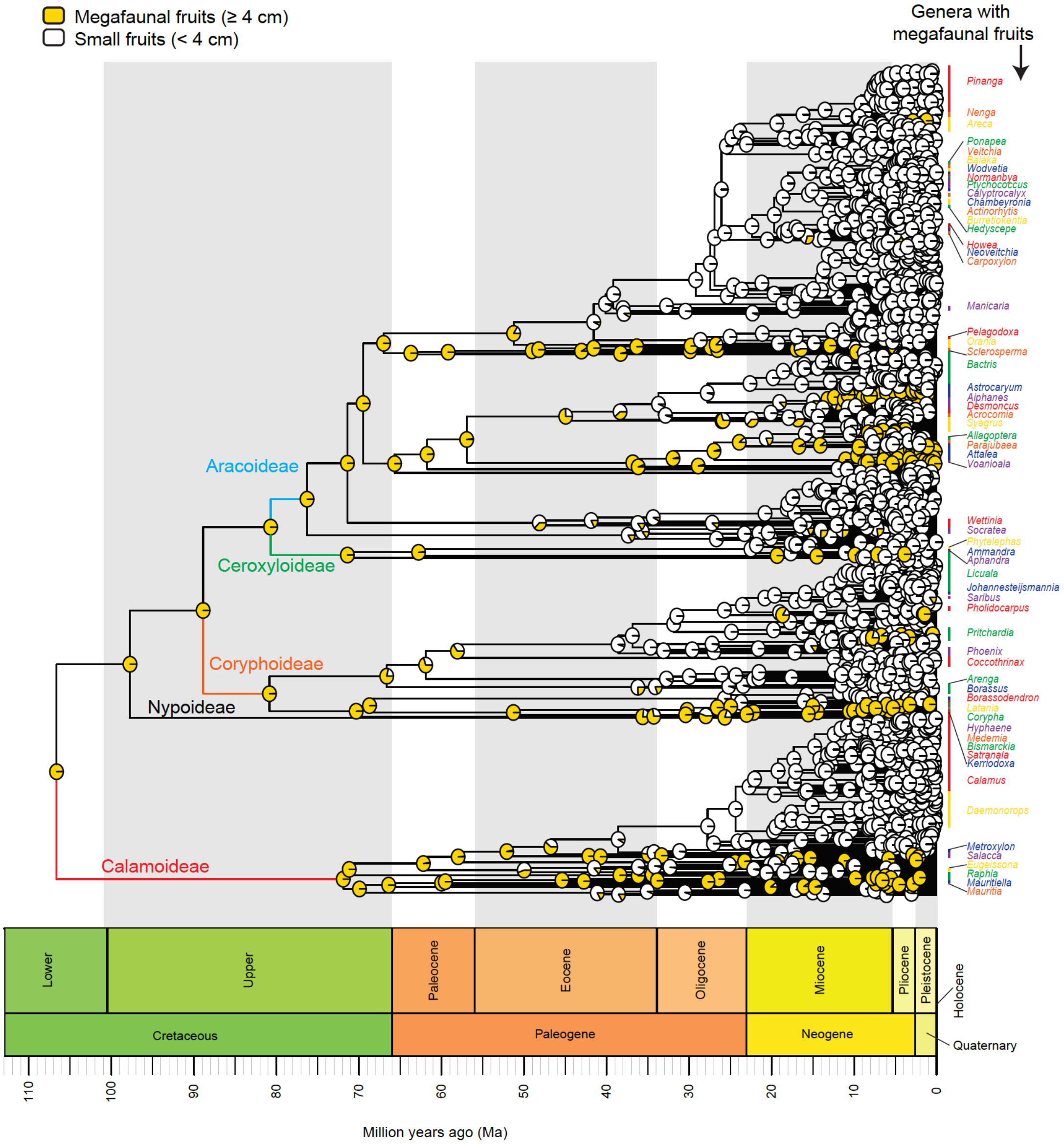

Figure S5: Lineage through time plots based on 100 empirical palm phylogenies (*n* = 1774, age = 105 Ma) and 100 simulated birth-death phylogenetic trees (*n* = 1774, age = 105 Ma) under speciation rate = 0.2 and extinction rate = 0.19. These simulated trees were used to evaluate whether the imbalance in tree shape, number of lineages and splitting events between the time slices influences the inference of extinction and / or transition rates through time (simulation results in figure S6). The black line refers to the Maximum Clade Credibility tree. This figure illustrates that the patterns of accumulation of lineages (overall shape) in the simulated data matches the empirical data quite well, particularly in the most recent time slices (i.e. last 10 Ma).

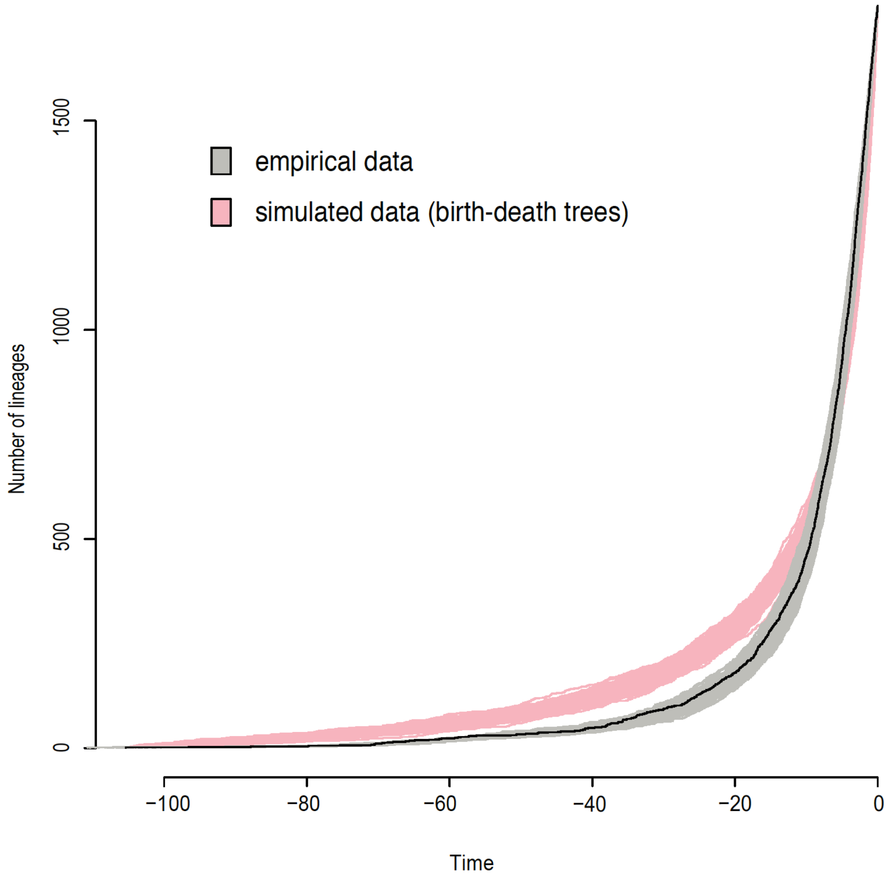

Figure S6: Posterior densities (box-and-whiskers) of time-dependent speciation (a), extinction (b) and transition (c) rates resulting from Markov chain Monte Carlo BiSSE on 10 simulated birth-death trees. Box-and-whiskers indicate the median, quartiles (25% and 75%), minimum (5%), maximum (95%) and outliers of these rates for each time slice over the last 25 my. Trees were simulated under speciation rate = 0.2, extinction rate = 0.19; and neutral binary traits were optimized on the trees under transition rate = 0.02. These results show that the time-dependent BiSSE model with time slices ranging from the distant (25 Ma) to the recent (0.5 Ma) past allows us to correctly infer speciation, extinction and transition rates, and the imbalance in tree shape, number of lineages and splitting events between the time slices does not necessarily influence the inference of extinction or transition rates. The accuracy of the extinction and transition rate estimates, however, does decrease towards more recent time slices, in which posterior densities are wider. This suggests that the increasing Quaternary extinction and transition rates observed from the empirical data (figure 3 in main text) are reliable.

a. Speciation rate b. Extinction rate c. Transition rate

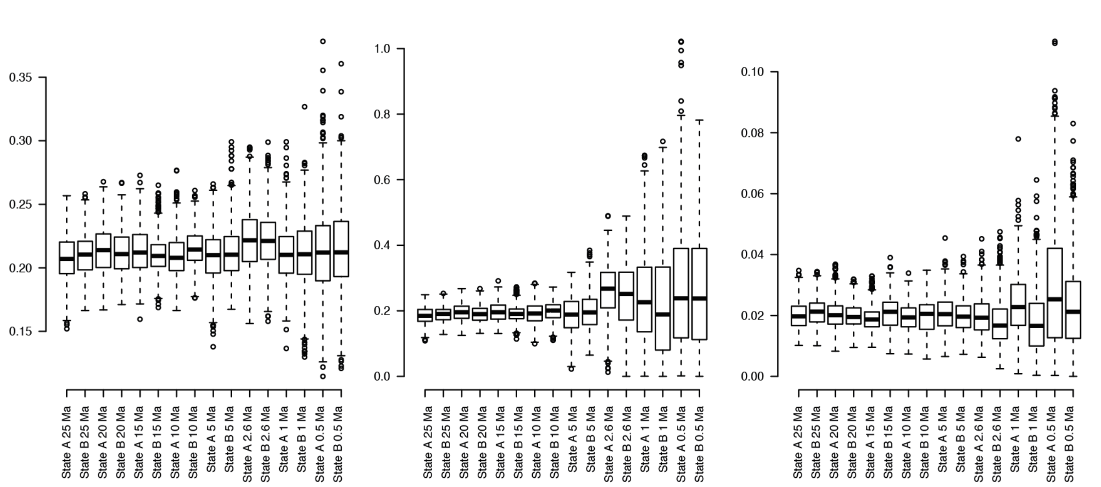

Figure S7: Posterior densities (box-and-whiskers) of time-dependent speciation (a, d), extinction (b, e) and transition (c, f) rates resulting from Markov chain Monte Carlo BiSSE for the simulated trait-dependent diversification process in which extinction (a–c) or transition (d–f) rates for trait state B were modeled to increase at 2.6 Ma. Box-and-whiskers indicate the median, quartiles (25% and 75%), minimum (5%), maximum (95%) and outliers of these rates for each time slice over the last 25 my. These results show that we have the power to reliably infer the modeled extinction rate shift (in state B, b) and transition rate shift (from state B to state A, f) from 2.6 Ma onwards, without erroneously inferring Quaternary increases in speciation (a, d) or transition rates (c). However, when modeling a transition rate shift (f), the extinction rate may also erroneously increase (e), suggesting that the increased extinction rate for megafaunal-fruited palms may be unreliable and simply result as an effect of increased transition rates. Nevertheless, in New World palms we detected increased extinction rates without also detecting increased transition rates (see figures 3b and 3e), suggesting that the increased extinction in New World palms may be a true phenomenon, as it does not result as an effect of increased transition rates.

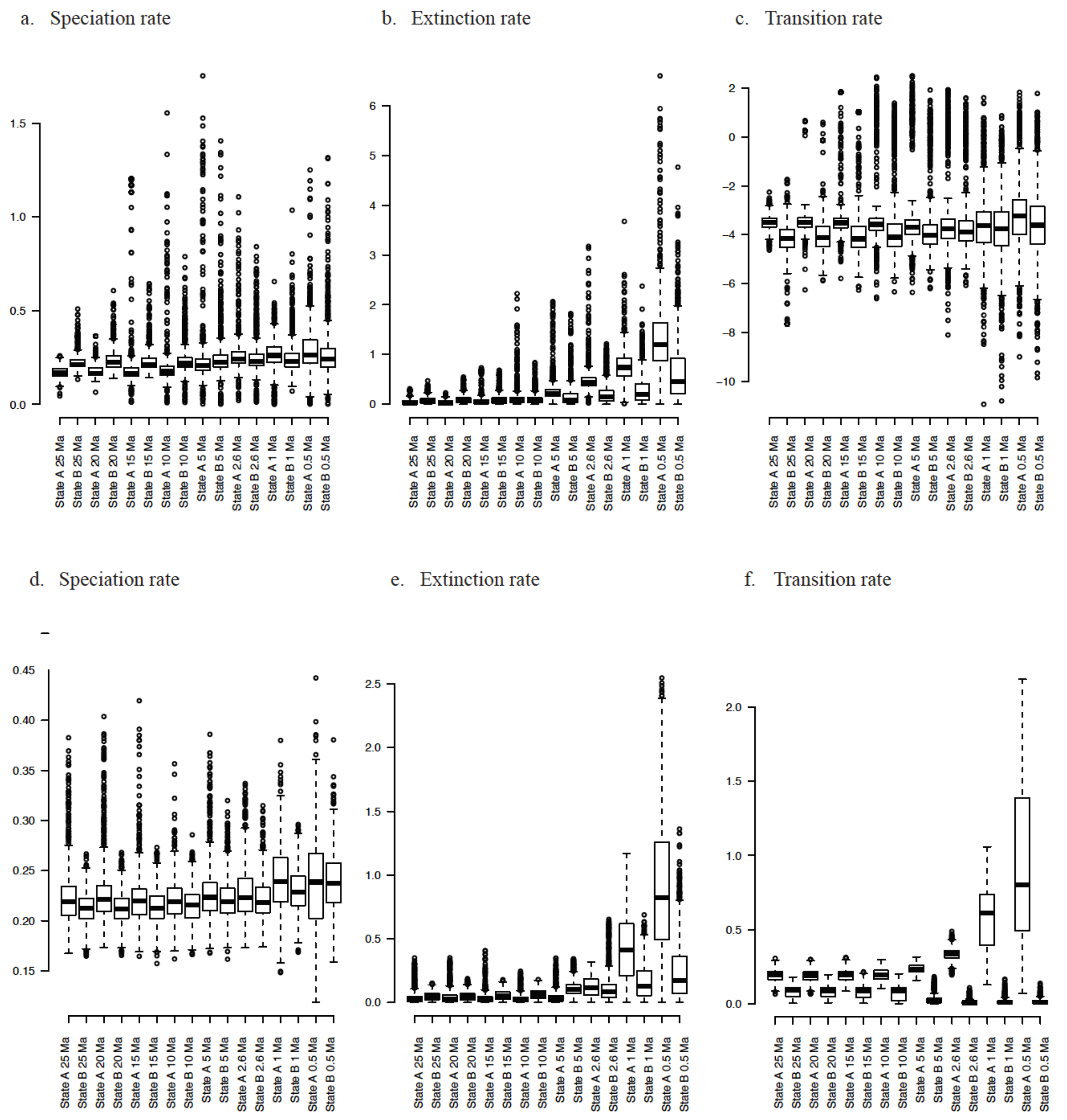

Figure S8: Parameters in the trait- and time-dependent Binary State Speciation and Extinction (BiSSE) models. Model selection was performed by constraining speciation, extinction and transition rate parameters between pre-Quaternary / Quaternary and / or between small- / megafaunal- fruited palm lineages. From the 45 models tested, the simplest model (with the fewest number of parameters and thus most constraints) without significantly affecting model fit based on likelihood-ratio tests and the Akaike information criterion (AIC) was selected (see tables S6–S8). λ = speciation rate, μ = extinction rate, q = transition rate. a-h refer to the different lineage-specific rates, e.g. λ_a is the speciation rate of pre-Quaternary small-fruited palm lineages.

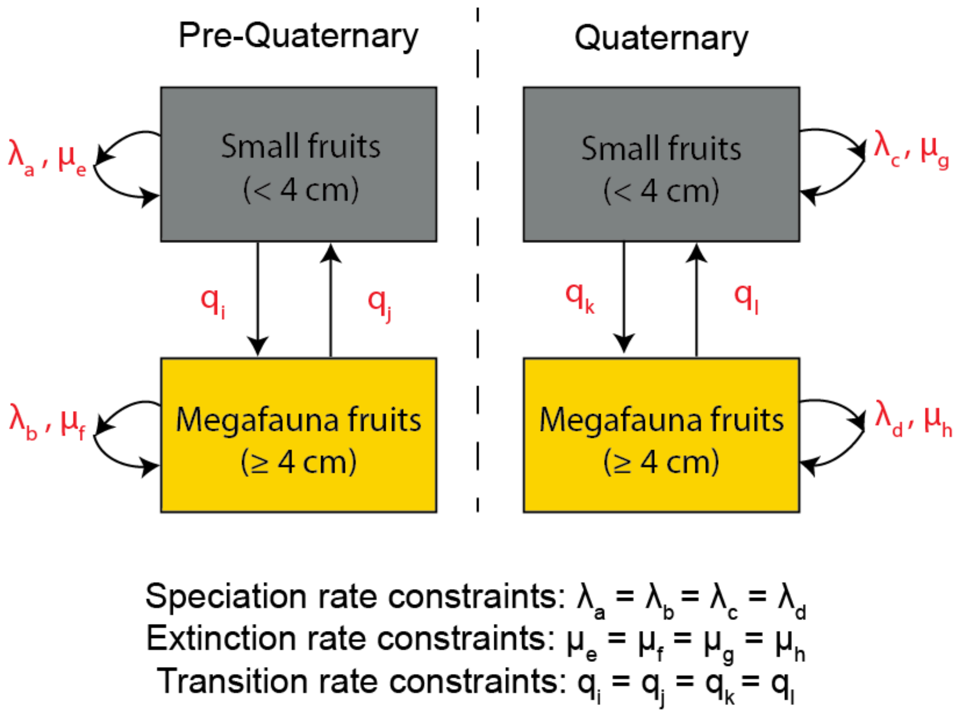

Figure S9: Posterior densities (box-and-whiskers) of time-dependent speciation (a, c, e), and net diversification (b, d, f) rates resulting from Markov chain Monte Carlo BiSSE for global (a, b), New World (c, d) and Old World (e, f) palms. Box-and-whiskers indicate the median, quartiles (25% and 75%), minimum (5%), maximum (95%) and outliers of these rates for each time slice over the last 25 my. Small-fruited palms have higher speciation rates than megafaunal-fruited palm lineages in global (a) and Old World (e) palms, consistently through time, whereas New World palms do not show such difference (d). Net diversification rates of megafaunal-fruited global (b) and New World (d) palms are rapidly decreasing since the Quaternary (2.6 Ma), as compared to small-fruited palms. In the Old World (f) net diversification rates of megafaunal- and small-fruited palm lineages have remained similar, and relatively constant through time.

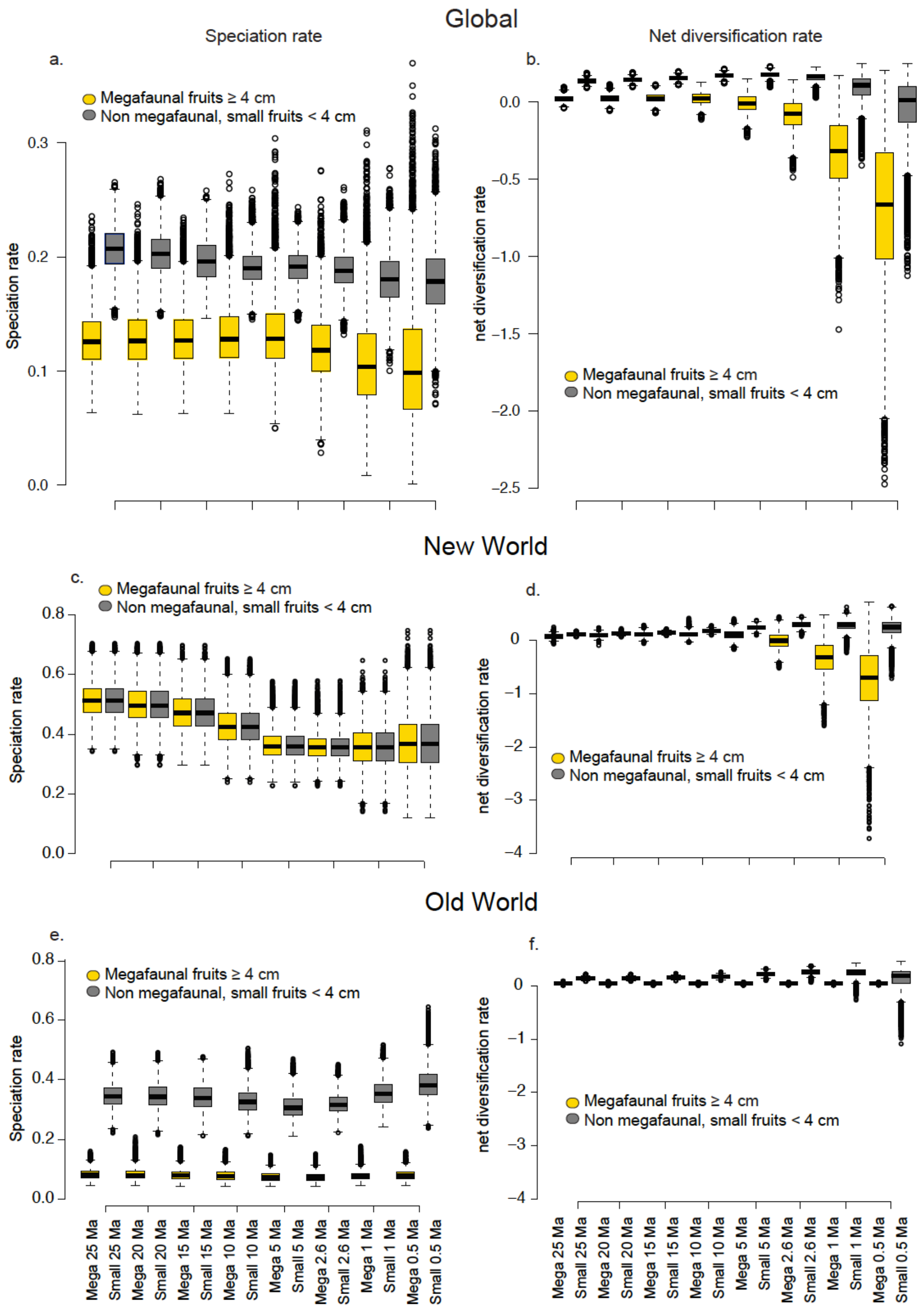

Figure S10: Posterior densities (box-and-whiskers) of time-dependent extinction (a, c) and transition (b, d) rates resulting from Markov chain Monte Carlo BiSSE for global palms when classifying megafaunal fruits as those ≥ 3.5 cm (a, b) or ≥ 4.5 cm (c, d). Box-and-whiskers indicate the median, quartiles (25% and 75%), minimum (5%), maximum (95%) and outliers of these rates for each time slice over the last 25 my. Similar to the original results (compare to figure 3 in the main text), in which megafaunal fruits were classified as those ≥ 4 cm, palms with megafaunal show increasing extinction rates (a, c) as well as increasing transitions towards small fruits (b, d) during the Quaternary (last 2.6 Ma).

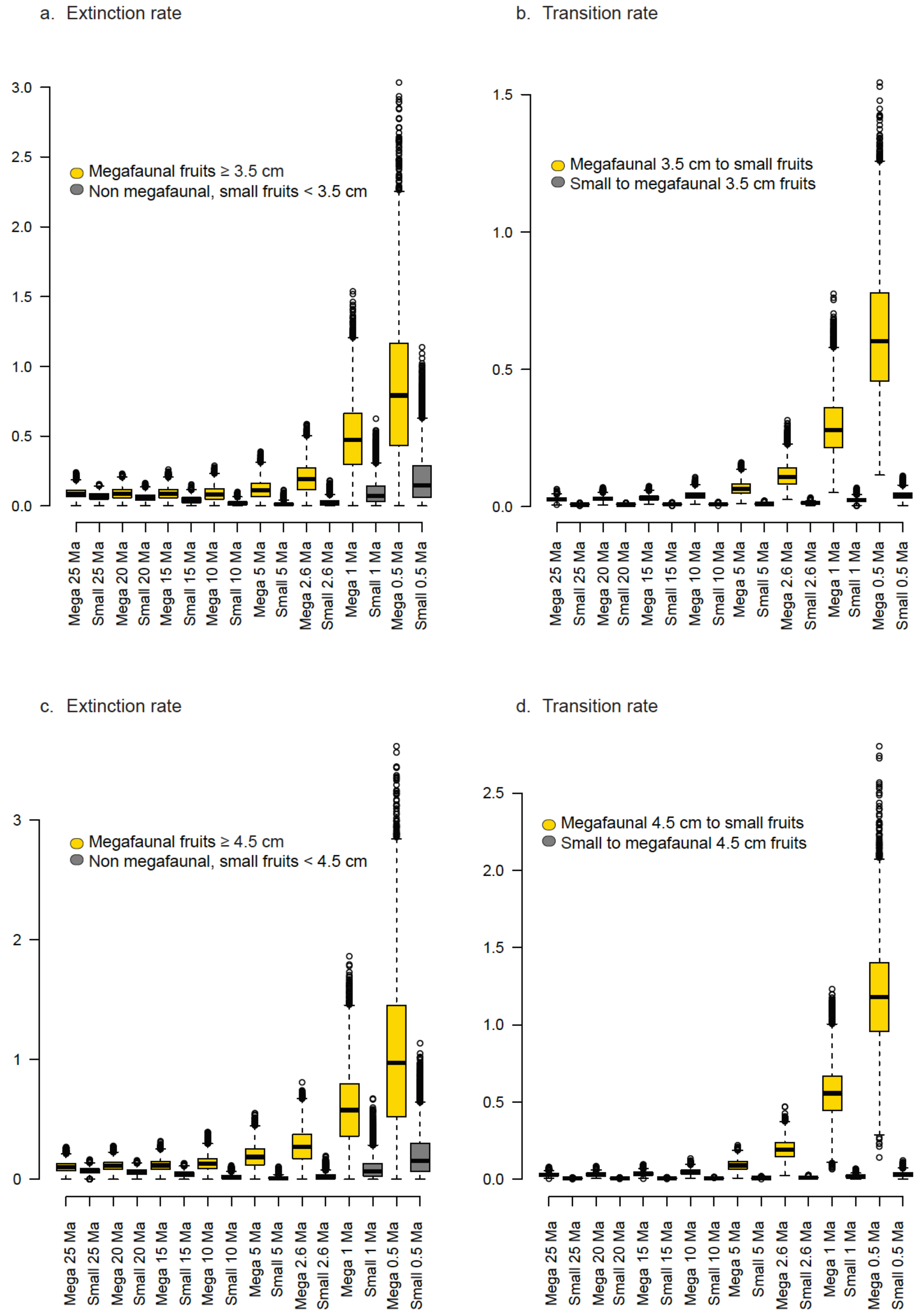

Figure S11: Posterior densities (box-and-whiskers) of time-dependent extinction (a) and transition (b) rates resulting from Markov chain Monte Carlo BiSSE for global palms when excluding subtribe Attaleinae from the data. Box-and-whiskers indicate the median, quartiles (25% and 75%), minimum (5%), maximum (95%) and outliers of these rates for each time slice over the last 25 my. Similar to the original results (compare to figure 3 in the main text), palms with megafaunal show increasing extinction rates (a) as well as increasing transitions towards small fruits (b) during the Quaternary (last 2.6 Ma).

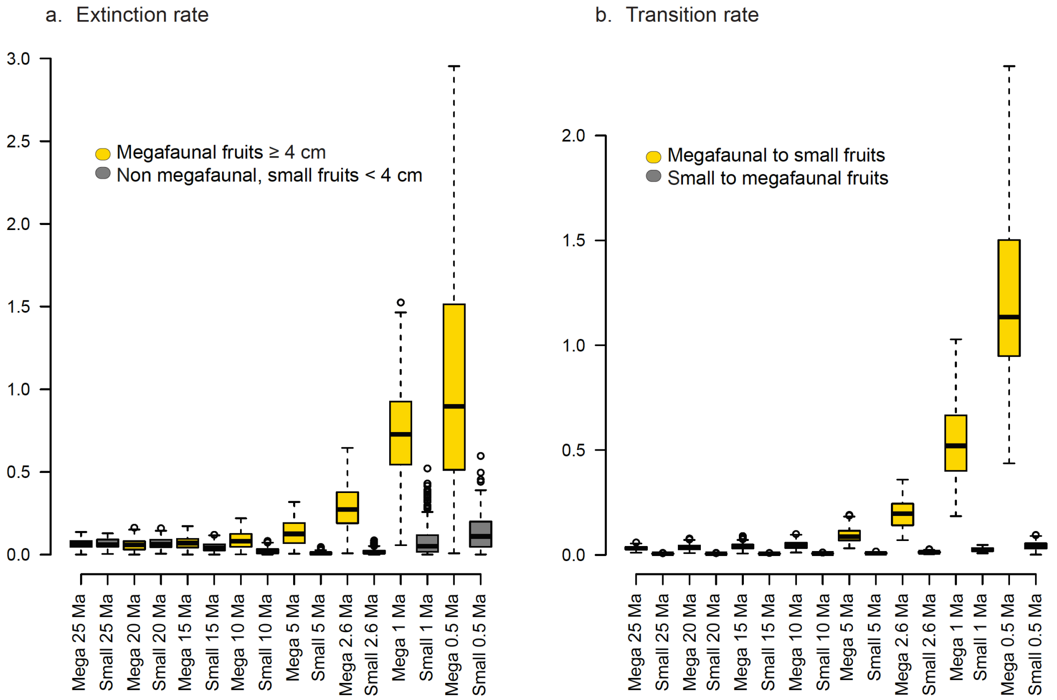

Supplement: Onstein_et_al_methods_tables_figures_ESM_R2 [file rspb20180882supp1.pdf]
