# Supplementary material for: To adapt or go extinct? The fate of megafaunal palm fruits under past global change
Source: Proc Biol Sci. 2018 Jun 13;285(1880):20180882. doi: 10.1098/rspb.2018.0882 (PMC6015859; doi:10.1098/rspb.2018.0882)
Supplement: ReadMe [file rspb20180882supp2.docx]

This is an R program that simulates a trait-dependent diversification process with a shift in rates at a given point in time.

It includes two R scripts:

- functions_for_simulation.R
- main_runSimulation.R

When using this program, please cite Onstein et al. “To adapt or go extinct? The fate of megafaunal palm fruits under past global change” in Proceedings of the Royal Society B, 2018.

For questions, contact Leonel Herrera-Alsina: [leonelhalsina@gmail.com](mailto:leonelhalsina@gmail.com)

Features:

1. It can handle trait-dependent speciation or trait-dependent extinction.

2. It is possible one single shift in rate regime at a given point in time.

3. It can only handle two traits.

4. This shift can comprise a change in speciation, extinction, transition rate or any combination. It is possible to specify that one trait changes and the other remains the same.

5. It produces phylogenetic trees along with trait states of extant lineages.

6. It is possible to specify the root state.

To keep in mind:

The simulation is a success when A) the crown lineages (species from the first split) survived to the present. So, it is suggested to consider the conditioning on survival of the crown lineages when setting the ML search and B) both trait states are present.

The program will run until the number of simulations are completed. Under certain parameter combinations, it might take very long until the first simulation is successful. This could happen because 1) speciation rate is just slightly larger than extinction which causes either extinction of the whole clade or extinction of the crown lineages, 2) one of the traits is very unlikely to make it to the present (because low transition rate to this state or an overall low diversification rate of it), 3) high diversification rates and large crown age would produce a massive number of species, the number of species will keep increasing and at some point, the trees would be difficult to handle. Because of this, I have set a condition that the simulation would stop when the limit in species (20,000) is reached. Otherwise it would keep increasing forever. Notice that these three circumstances are normal in a birth-death model.

As an example, I ran a simulation of a 30 million years old clade, with constant lambda across time and traits (0.2), uniform transition rate of 0.02. The shift in rate regime only affected extinction on trait 2, which switched dramatically from 0.02 to 0.3. The other trait kept the same rates (0.02). The shift took place 2 million years before the present. My laptop performed 10 simulations in 18 seconds.

I recommend to start with 1 simulation and switch to TRUE the argument see_progress in order to see what the behavior of the chosen parameter is. Package DDD is required.
